# Identification of Cell Cycle Dependent Interaction Partners of the Septins by Quantitative Mass Spectrometry

**DOI:** 10.1371/journal.pone.0148340

**Published:** 2016-02-12

**Authors:** Christian Renz, Silke Oeljeklaus, Sören Grinhagens, Bettina Warscheid, Nils Johnsson, Thomas Gronemeyer

**Affiliations:** 1 Ulm University, Department of Molecular Genetics and Cell Biology, Ulm, Germany; 2 University of Freiburg, Department of Biochemistry and Functional Proteomics, Institute of Biology II, Faculty of Biology and BIOSS Centre for Biological Signalling Studies, Freiburg, Germany; Dartmouth College, UNITED STATES

## Abstract

The septins are a conserved family of GTP-binding proteins that, in the baker's yeast, assemble into a highly ordered array of filaments at the mother bud neck. These filaments undergo significant structural rearrangements during the cell cycle. We aimed at identifying key components that are involved in or regulate the transitions of the septins. By combining cell synchronization and quantitative affinity-purification mass-spectrometry, we performed a screen for specific interaction partners of the septins at three distinct stages of the cell cycle. A total of 83 interaction partners of the septins were assigned. Surprisingly, we detected DNA-interacting/nuclear proteins and proteins involved in ribosome biogenesis and protein synthesis predominantly present in alpha-factor arrested that do not display an assembled septin structure. Furthermore, two distinct sets of regulatory proteins that are specific for cells at S-phase with a stable septin collar or at mitosis with split septin rings were identified.

Complementary methods like SPLIFF and immunoprecipitation allowed us to more exactly define the spatial and temporal characteristics of selected hits of the AP-MS screen.

## Introduction

The baker’s yeast *Saccharomyces cerevisiae* undergoes asymmetric cell division by budding.

Establishment and maintenance of cell polarity requires a septin scaffold at the bud neck that attracts different protein complexes during the cell cycle [1]. Yeast cells express five septins during vegetative growth, namely Cdc3, Cdc10, Cdc11, Cdc12 and Shs1. They assemble into hetero-oligomers by association of monomers with the order Cdc11-Cdc12-Cdc3-Cdc10-Cdc10-Cdc3-Cdc12-Cdc11 with Shs1 sometimes replacing the terminal subunit Cdc11 [2,3].

The septin filaments assemble at the bud neck in an organized array, the so-called septin ring. This septin ring undergoes different cell cycle-dependent architectural transitions [4]. In early G_1_-phase, the septins are recruited to and accumulate at the presumptive bud site in a patch-like structure. Shortly before bud emergence, the patches are transformed into a ring marking the future site of bud growth and cytokinesis [5]. After bud formation, the septin ring expands into a stable hourglass-shaped collar that is present at the bud neck until the onset of mitosis. Before cytokinesis, the septin collar splits into two distinct rings, one located at the mother and one at the daughter side of the bud neck [4]. Cell separation then occurs between the two rings. After completion of cell separation, the old septin rings are disassembled and septin subunits are partially replaced and recycled for the next round of the cell cycle [6].

The initial recruitment of the septins to the future bud site depends on the small GTPase Cdc42, its effectors Gic1 and Gic2, and the action of the cyclin-dependent kinases Cdc28 and Pho85 [7]. Septin filament assembly is supposed to be mediated by the diffusion-driven annealing of the septin rods at the plasma membrane [8]. The transition of the septin ring into a stable septin collar after bud emergence is associated with the phosphorylation and acetylation of certain subunits [9–12]. Splitting of the septin collar at the onset of cytokinesis is supposed to be initiated by a collective switch in the orientation of the septin filaments from parallel to perpendicular to the growth axis of the cell [13,14]. The switch is accompanied by at least two different modifications. First, the bud neck kinase Gin4 phosphorylates Shs1 at residues different from those being modified in G_1_-phase [15]. Second, the small ubiquitin-like modifier (SUMO) Smt3 is covalently attached to Cdc3, Cdc11 and Shs1 at the mother side of the bud [16].

A deeper understanding of the regulation of septin structure assembly and subsequent structural transitions requires the identification of all involved molecular components as well as a temporal map of their post-translational modifications and of their dynamic organization into multi-protein complexes. However, systematic interaction screens have yet only been performed for mammalian septins were cDNA libraries were screened for SEPT protein baits using the yeast two hybrid system [17]. The interactome of the yeast septins remains incomplete and rests on numerous targeted studies that aimed at deciphering a certain function or process [18].

We tried to address this shortcoming by systematically screening for specific septin interactors at distinct stages of the cell cycle. Affinity purification followed by mass spectrometry (AP-MS) has evolved as a very efficient tool to identify protein-protein interactions [19,20]. To allow for a spatial and temporal integration of the resulting interaction networks into the context of the cell and its different cell cycle states, we have combined cell synchronization with AP-MS. Synchronization of yeast cells in cell cycle states where septins have a distinct structure (patch—collar—split rings) was followed by affinity purification of tagged septin complexes and SILAC based quantitative mass spectrometry (MS) analysis. This unique combination allowed us to describe the changing pattern of interaction partners of the septins during the cell cycle.

Selected candidates were subjected to a SPLIFF (**Spli**t Ubiquitin **F**luorophore **F**luorophore) analysis [21] to better resolve spatial and temporal aspects of these interactions. The integration of both techniques allowed us to systematically explore the cellular aspects of protein interactions detected by AP-MS.

## Methods

### Plasmids and strains

The construction of the yeast strains used in this study is described in detail in the Supporting Information.

All yeast strains used in this study are listed in the S3 Table.

### Growth of yeast strains and cell synchronization

The Cdc11-TAP strain was grown in standard SD medium and the Cdc11-GFP strain in SILAC-SD medium containing ^13^C_6_,^15^N_4_-L-arginine and ^13^C_6_,^15^N_2_-L-lysine (Sigma Aldrich) as only source for arginine and lysine. Incorporation of labeled amino acids was confirmed by analyzing protein extracts from labeled cells by gel-based nano-HPLC-ESI-MS/MS analysis.

Full incorporation of the labeled amino acids was obtained by diluting a preculture grown overnight in SILAC medium into fresh SILAC medium and incubating it at 30°C for 7.5 h (S1 Fig).

An overnight culture of each strain was diluted into 400 ml fresh medium and grown overnight at 30°C to the desired OD_600nm_ for cell synchronization. The synchrony of the cultures was monitored by microscopy and the cells were harvested by centrifugation and immediately used for affinity purification.

Cell synchronization was performed either with alpha-factor (Sigma) (3.7 μM final concentration pre-dissolved in 0.1 M HCl; added to the cell culture at an OD_600nm_ of 1 and incubated at 30°C for 2:40 h) or hydroxyurea (Sigma) (0.2 M final concentration; added to the cell culture at an OD_600nm_ of 1 and incubated at 30°C for 3:30 h). Temperature-sensitive strains were arrested by incubation at 37°C for 3:25 h after the OD_600nm_ reached 0.6.

### Affinity purification (AP)

AP was performed separately for the Cdc11-TAP- and the Cdc11-GFP-expressing control strain. The pelleted yeast cells were resuspended in extraction buffer (20 mM Tris pH 7.5, 80 mM NaCl, 1 mM DTT, 8.3 μM antipain, 0.3 μM aprotinin, 1 mM benzamidine, 1 μM bestatin, 10 μM chymostatin, 1.5 μM pepstatin A, 5 μM leupeptin, 1 mM PMSF, 10 mM β-glycerophosphate, 10 mM NaF, 1 mM sodium orthovanadate), acid washed glass beads were added (3-fold wet cell weight) and twelve cycles of bead beating were applied (1 min vortexing followed by 1 min incubation on ice). The resulting extract was clarified by centrifugation for 15 min at 40,000 x g. The protein concentrations were adjusted to 5 mg/ml in extraction buffer and 10% (v/v) glycerol was added. CNBr activated Sepharose (GE Healthcare) was coupled with human IgG (MP Biomedicals) according to the manufacturer's protocol. The obtained HsIgG-coupled Sepharose was equilibrated in extraction buffer and 75 μl slurry per 50 mg protein were added to the protein extract.

The samples were rotated overhead overnight at 4°C and subsequently another equivalent of hsIgG-sepharose was added followed by an incubation for 2 h at 4°C. The Sepharose was washed with a 50-fold hsIgG-Sepharose slurry volume of extraction buffer (containing 10% (v/v) glycerol and lacking aprotinin, benzamidine and NaF) and bound protein was eluted with 6his-AcTEV Protease (Life Technologies). The protease was removed using Ni-NTA agarose. Supernatants containing the cleaved protein complexes from both strains were mixed. Proteins were precipitated by adding the 4-fold volume of ice-cold acetone and stored for at least two days at -20°C. Precipitated proteins were collected by centrifugation, acetone was removed and the pellets were resuspended in a total volume of 40 μl 2x Lämmli buffer. Proteins were separated by SDS-PAGE followed by colloidal Coomassie blue staining.

Samples from all steps of the purification were submitted to Western blot analysis using anti-Protein A (Sigma) and anti-GFP (Roche) primary antibodies.

### Mass spectrometry and data analysis

Following gel electrophoresis of affinity-purified, differentially SILAC-labeled Cdc11-complexes and colloidal Coomassie blue staining, gel lanes were cut into individual slices. The gel slices were destained and washed as described [22]. Cysteine residues were reduced using 10 mM DTT (30 min at 65°C) and alkylated with 50 mM iodoacetamide (30 min at room temperature in the dark). Proteins were subsequently in-gel digested using trypsin (37°C, overnight). Peptide mixtures of three independent replicates per cell cycle arrest were analyzed by LC/MS using an UltiMate 3000 RSLCnano HPLC system (Thermo Scientific) coupled to an LTQ-Orbitrap XL mass spectrometer (Thermo Scientific).

Mass spectrometric data were processed using the software MaxQuant [23] (version 1.2.0.18 for alpha-factor and hydroxyurea data or 1.3.0.5 for *cdc15-1* data). For protein identification, MS/MS data were searched against the *Saccharomyces* Genome Database (SGD; www.yeastgenome.org; version of June 2012) using Andromeda [24]. The detailed search engine parameters are given in the Supporting Information. The mass spectrometry proteomics raw data and MaxQuant result files have been deposited to the ProteomeXchange Consortium [25] via the PRIDE partner repository with the dataset identifier PXD002561.

Light-over-heavy (L/H) abundance ratios for proteins were log_10_-transformed and mean log_10_ L/H ratios across all three biological replicates of septin complexes purified from yeast cells treated with alpha-factor or hydroxyurea and from *cdc15-1* mutant cells as well as p-values (one-sided Student's t-test) were determined. Proteins with a p-value of < 0.05 and a mean L/H ratio of > 4 were considered as putative Cdc11-interacting proteins. All quantified proteins are listed in the S1 Table. Data were visualized by plotting the mean log_10_ ratios of all proteins quantified in at least two replicates *versus* the negative value of the corresponding log_10_-transformed p-value. Candidates were finally sorted into manually defined categories according to their functions that were retrieved from the SGD.

Proteins known to be mislocalized under the respective blocking conditions were excluded from the subsequent candidate selection and evaluation process. See the Supporting Information for details.

### Microscopy and SPLIFF analysis

All microscopy experiments were performed on an Axio Observer SD spinning disc confocal microscope (Zeiss) equipped with an Evolve512 EMCCD camera (Photometrics), objectives Plan-Apochromat 63X/1.4 Oil DIC and Plan-Apochromat 100X/1.4 Oil DIC (Zeiss), and 488 nm and 561 nm diode lasers (Zeiss). The microscope was controlled by the ZEN2012 software package (Zeiss). Images were analyzed and created using ZEN2012 or ImageJ64 (version 1.48p).

For microscopy of synchronized yeast cells, a saturated overnight culture of a BY-SILAC strain expressing Shs1-mCherry and a candidate-GFP fusion was diluted into fresh medium and incubated overnight at 30°C until the desired OD_600nm_ was reached. The cell cycle was arrested using the protocols described above. Arrested cells were concentrated by brief centrifugation, loaded onto a microscopy glass slide, fixed with a glass coverslip and directly observed by fluorescence microscopy.

For time lapse mircoscopy, cells from an exponentially growing culture were mounted onto an agar pad prior to microscopy. Microscopy was performed at 30°C and images were acquired every 3 min for up to 5 h. Time resolved SPLIFF analysis was performed as described elsewhere [26].

For steady state SPLIFF analysis, a saturated overnight culture of diploid cells expressing Shs1-CCG and a N_ub_-fusion was diluted 1:10 into fresh medium containing 0 or 100 μM Cu^2+^ and grown for at least 4 h at 30°C. Cells were concentrated by brief centrifugation, loaded onto a microscopy glass slide, fixed with a glass coverslip and observed immediately. The microscope settings and data evaluation is described in the Supporting Information.

### Pulldown of candidate proteins using purified septin rods

The expression and purification of recombinant SNAP-tagged yeast septin rods was performed as described previously [27]. 6his-SNAP was purified from crude *E*. *coli* extracts with immobilized metal affinity chromatography followed by size exclusion chromatography as described for other proteins [27].

As a matrix for pulldown experiments, benzylguanine coupled to Sepharose (BG-Sepharose) was used. Briefly, CNBr-activated Sepharose 4B (GE Healthcare) was functionalized with BG-PEG-NH_2_ (NE Biolabs) (1 μmol per ml Sepharose) according to the Sepharose' manufacturer's recommendations. Remaining active groups were blocked for 3 h with 1 M ethanolamine pH 8.0.

Extracts of yeast cells expressing the respective TAP-tagged (or GFP tagged Vps1) candidate proteins (50 ml culture volume) were prepared as described above. Pulldown buffer (50 mM Tris pH 8.0, 300 mM NaCl) was used for preparing all subsequent protein solutions and washing steps. Septin rods and SNAP protein were captured onto BG-Sepharose (150 μl of 1 μM SNAP-tagged septin rods or 150 μl of 2 μM 6his-SNAP onto 60 μl BG-Sepharose slurry). The matrix was washed, blocked with 1 mg/ml BSA (1 h under rotation at 8°C) and subsequently incubated with the yeast extract (2 h under rotation at 8°C). The beads were washed five times and captured proteins were eluted by boiling the beads for 5 min in 40 μl of 2x Lämmli buffer. Eluted proteins were detected by Western blot analysis using an anti-protein A (Sigma) or anti-GFP (Roche) primary antibody.

## Results

### An experimental platform for the identification of cell cycle specific septin interacting proteins

To identify septin interacting proteins at different stages of septin architecture via AP-MS, a robust platform for cell synchronization and complex isolation was implemented in the first place. We constructed a yeast strain expressing the endogenous septin Cdc11 fused at its C-terminus to a TAP tag for the isolation of septin complexes on Protein-A Sepharose. As control strain, we constructed a strain expressing Cdc11-GFP. This strain allows additionally to monitor the state of septin architecture by fluorescence microscopy and to calculate the synchrony of the treated cells.

**Fig 1:**
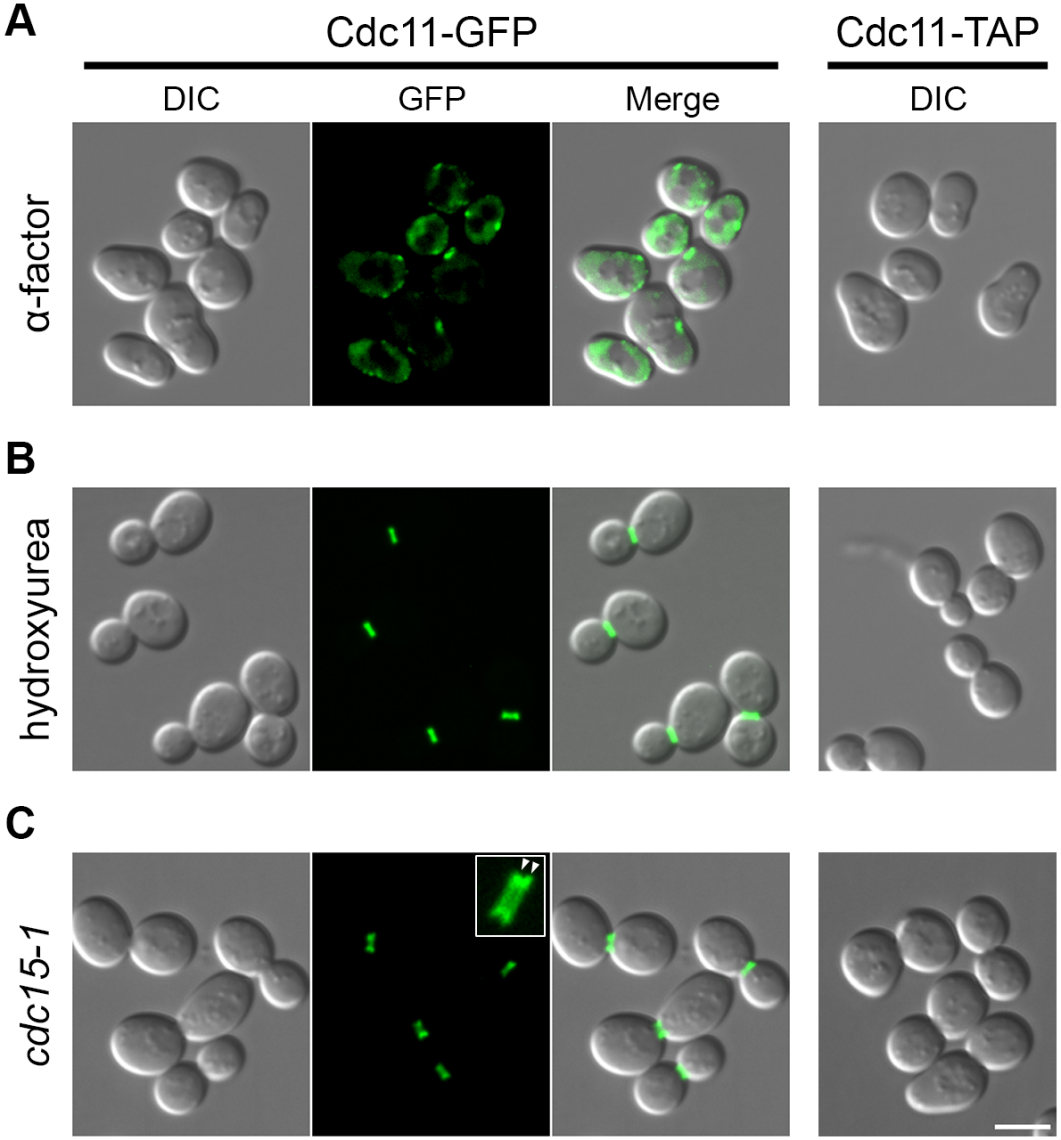
Cell synchronization of Cdc11-GFP and Cdc11-TAP expressing cells. Representative image sections (z-projection) for Cdc11-GFP or Cdc11-TAP expressing strains using the indicated blocking approaches. The structure within the white rectangle represents a zoom in of the respective septin architecture using a 100x objective. White arrowheads indicate split septin rings in *cdc15-1*-arrested cells. Scale bar 6 **μ**m.

We chose to investigate the protein composition of three different septins structures: (i) septin patches before their assembly into a distinct structure, (ii) the stable collar and (iii) the split septin rings. The first two states were realized by blocking the cells with alpha-factor (G1 phase) or hydroxyurea (HU) (S1 phase), respectively. Cells bearing the temperature sensitive allele *cdc15-1* arrest at the restrictive temperature in late anaphase [28]. Using the Cdc11-GFP strain, we confirmed that the septins are organized in patches at the membrane or are diffusely localized in the cytoplasm in alpha-factor arrested cells (Fig 1: top). HU blocked cells show the stable septin collar at the bud neck (Fig 1: middle), whereas *cdc15-1* arrested cells display the split septin rings (Fig 1: bottom). The synchrony of blocked cells was determined by counting the proportion of cells without an assembled septin ring for alpha-factor arrest (94.9 ± 0.6%; n ≥ 409 cells), with a stable septin collar for HU arrest (82.0 ± 5.4%; n ≥ 141 cells) and with split septin rings for *cdc15-1* arrest (85.6 ± 2.3%; n ≥ 162 cells). Synchronicity rates were calculated individually for each culture that was subsequently used in AP-MS-experiments. The cells were incubated with the respective blocking compound or at the non-permissive temperature and harvested for AP-MS in the blocked state.

We used a quantitative AP-MS approach to distinguish between genuine septin interacting proteins and contaminants. **S**table **i**soptope **l**abeling of **a**mino acids in **c**ell culture (SILAC) enables to distinguish in one MS experiment between protein complexes isolated from a target strain (here: Cdc11-TAP) from those isolated from a control strain (here: Cdc11-GFP) [29]. Briefly, one of the two strains (here: Cdc11-GFP) is labelled with "heavy", non-radioactive amino acid isotopes. Both strains are mixed and processed jointly for MS analysis. The control strain serves as a reference to identify non-specific binding to any of the used materials. In the case of such an unspecific binding, a contribution of "heavy" peptides from the control strain to an identification hit in the MS data analysis will be detected. Of importance is the stage where both strains are mixed: Mixing the cells or extracts of both strains before complex isolation ("purification after mixing") results in the identification of the stable core complex constituents whereas mixing of the eluates after purification allows also for the identification of transient interaction partners [30,31].

**Fig 2:**
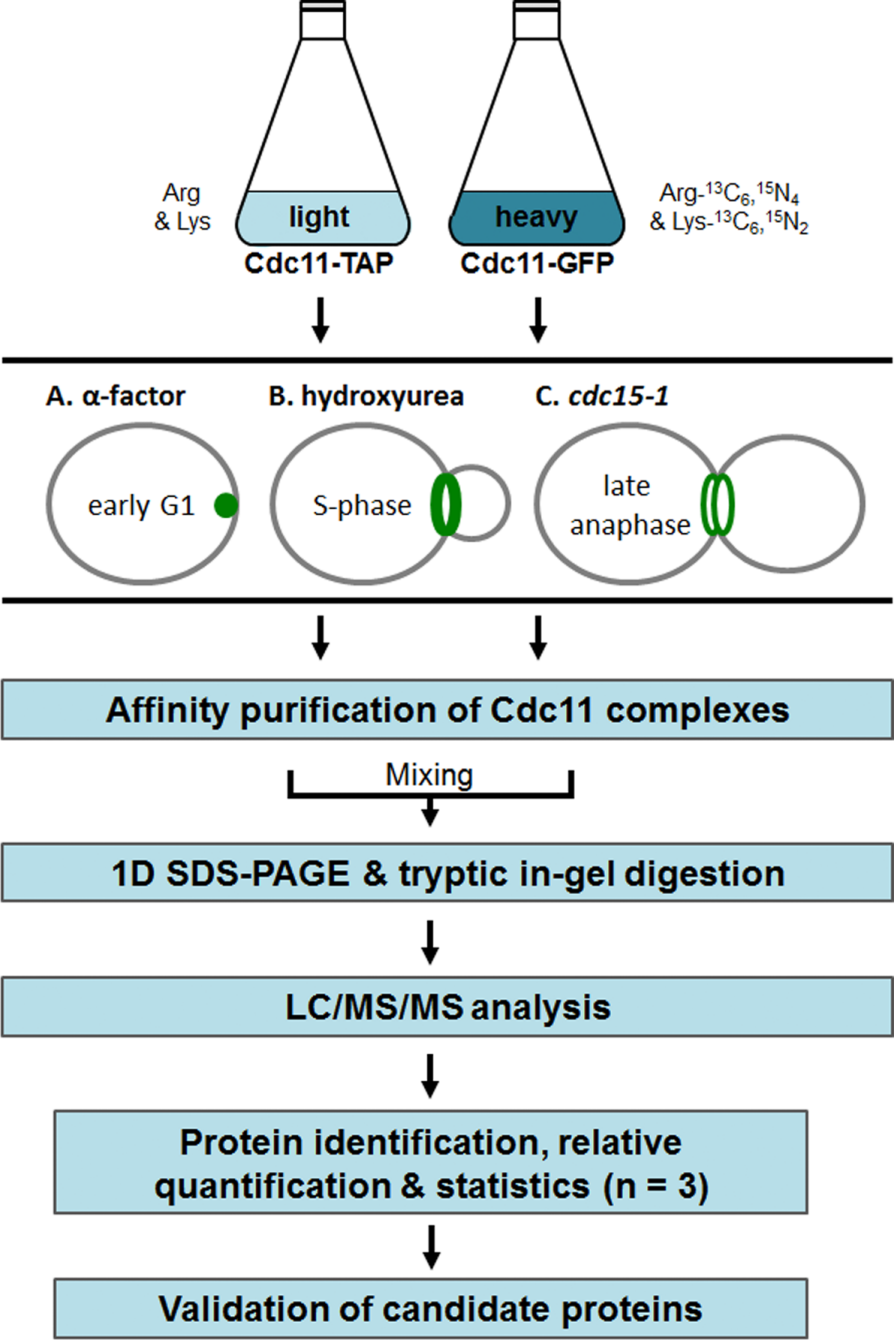
Workflow for SILAC-AP-MS. Yeast strains expressing Cdc11-TAP and Cdc11-GFP (metabolically labelled with ^13^C_6_,^15^N_4_-L-arginine and ^13^C_6_,^15^N_2_-L-lysine) are blocked with the indicated methods and subjected separately to affinity purification. The eluates are mixed and subjected to separation by SDS-PAGE followed by nano HPLC-ESI-MS/MS analysis and statistical data evaluation.

We have chosen a mixing after purification approach to monitor also transient interactions. Equal volumes of the eluates from the AP-MS were combined prior to SDS-PAGE and subsequent LC/MS analysis. The whole workflow is depicted in Fig 2. Affinity purifications were monitored by Western blot analysis of representative samples taken during the purification process (S2 Fig).

### Identification of septin interaction partners with AP-MS

**Fig 3:**
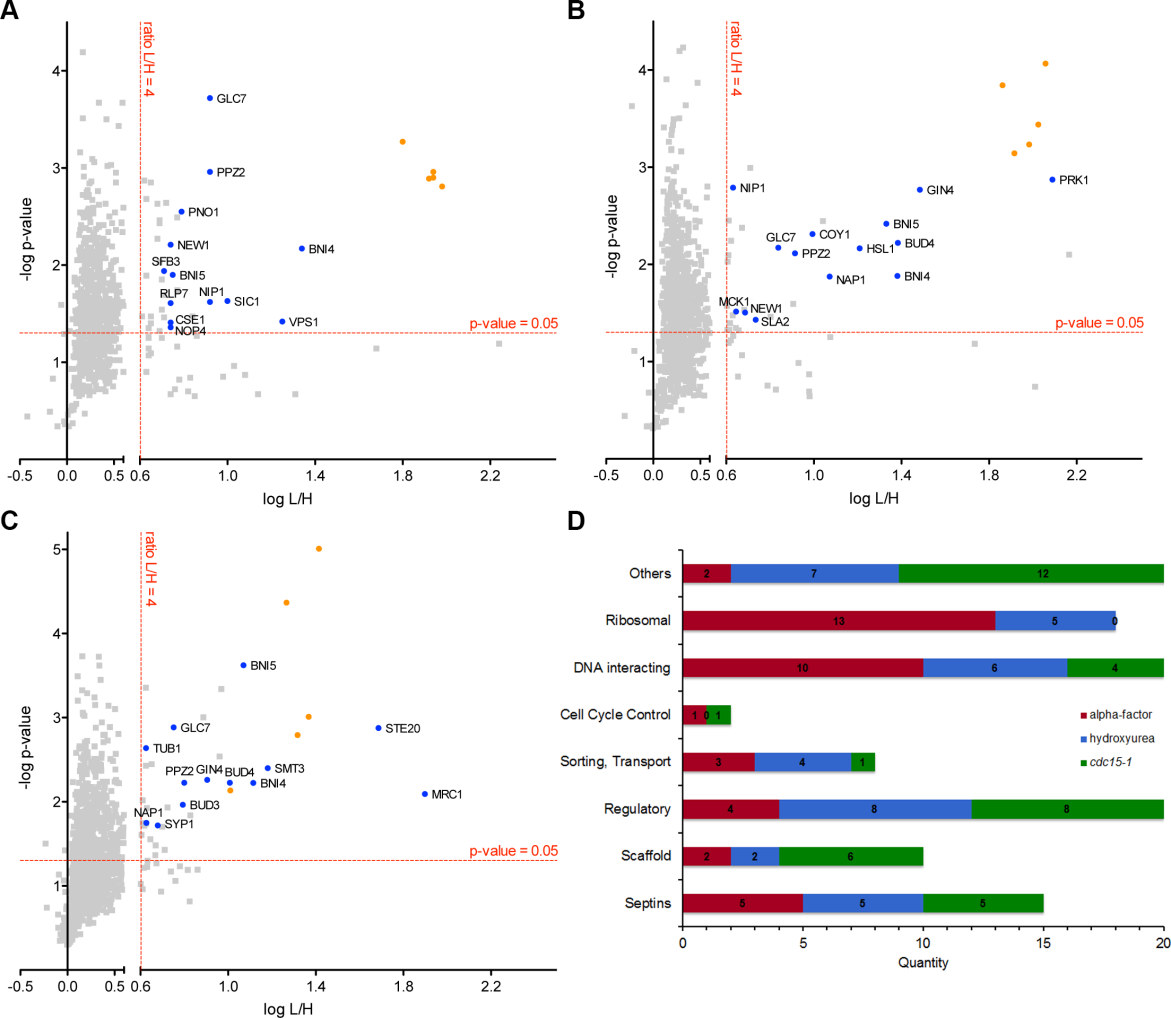
SILAC-AP-MS analysis. Results of the quantitative MS-analysis. Volcano plots for the MS analysis of cells arrested in G1 phase (alpha facor). (**A**), S-phase (**B**) or anaphase (**C**). The red-dashed lines indicate the thresholds defining a specific interaction. Selected candidate proteins for further validation are named and labeled in blue, septins are marked in red. Specific interactors were grouped into the indicated categories (**D**). Numbers represent the specific interactors in the respective timepoints for each category.

We performed three independent AP-MS purifications for each synchronization approach. “Light-over-heavy” (L/H) ratios and p-values (using a one-tailed t-test) were determined for each protein. The ratio L/H reflects the enrichment of a protein in the Cdc11-TAP purification (L) in comparison to the Cdc11-GFP purification (H). The negative logarithm of the p-value was plotted against the logarithm of the L/H ratio (Fig 3). Proteins quantified in at least two out of three replicates and exhibiting a ratio ≥ 4 and a p-value < 0.05 were considered as specific interaction partners. All quantified proteins are listed in the S1 Table.

The specific interaction hits were sorted into the following manually defined categories: Septins, scaffold proteins, regulatory proteins, proteins involved in intracellular trafficking, cell cycle control proteins, DNA interacting proteins, ribosomal proteins, others not fitting in one of these categories.

A total of 1,209 proteins were quantified for alpha-factor synchronized cells. In this dataset, 40 proteins (including the five mitotic septins) fulfill the criteria for specific septin interaction partners (Fig 3A). In alpha-factor arrested cells, the identified septin interactors are predominantly proteins involved in ribosome biogenesis or protein synthesis (13 proteins, 32.5%) and nuclear proteins (10, 25.0%). The other defined categories are illustrated in Fig 3D.

In HU arrested cells, a total of 1,446 unique proteins were quantified. Only 37 of these hits (including the five mitotic septins) fulfill the specificity criteria and were assigned as S-phase specific interactors of the septins (Fig 3B). Among the interactors are eight regulatory proteins such as protein kinases. The number of identified ribosomal and nuclear proteins is significantly reduced. A classification of all interactors is shown in Fig 3D.

A total of 1,278 unique proteins were quantified in cells arrested in late anaphase. Of these, 37 (including the five mitotic septins) were assigned as specific interaction partners (Fig 3C). Besides regulatory and scaffold proteins and proteins involved in processes formerly not associated with the septins (glucose and glycerol metabolism, protein folding, membrane organization), Syp1, a protein involved in endocytosis and septin organization [32] and Mrc1, a protein required for cell cycle control [33] were identified as specific interaction partners of the septins. The classification of all anaphase specific interaction partners is shown in Fig 3D.

The overlaps of the three replicates evaluated for each time point are illustrated in S3 Fig.

**Fig 4:**
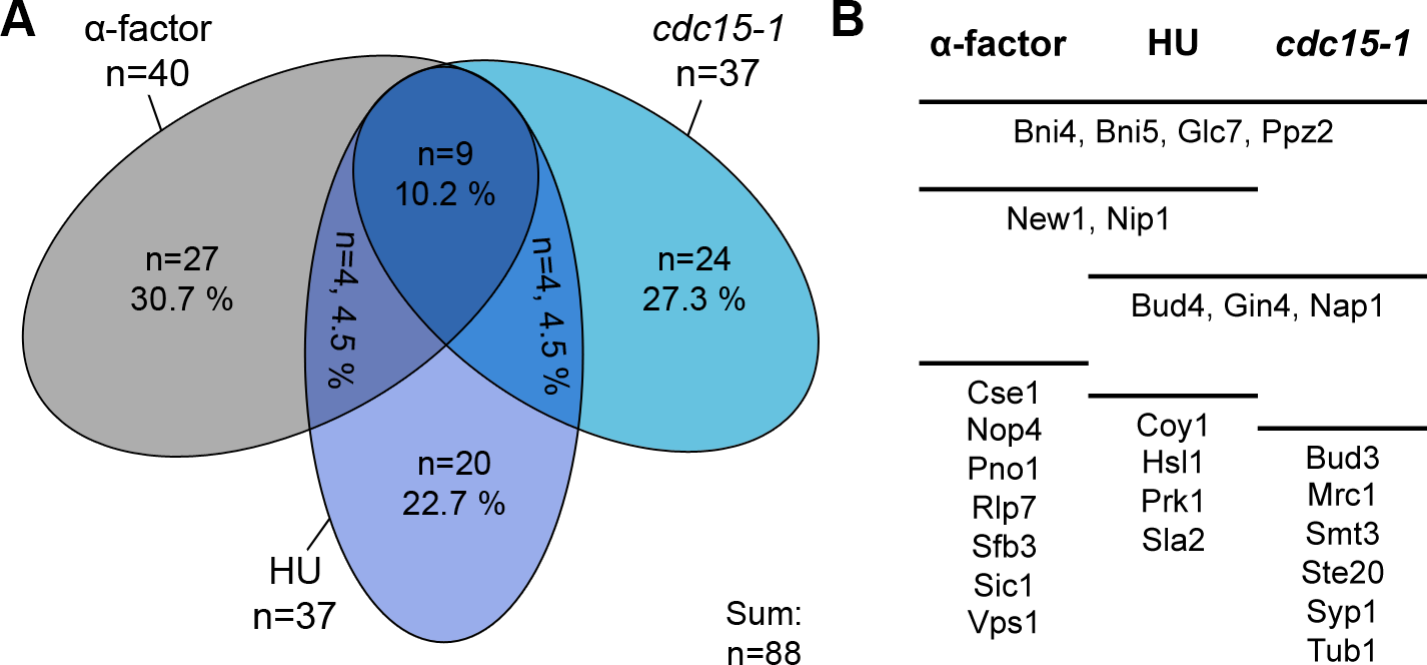
Overview of the SILAC-AP-MS datasets. **(A)** Overlap of the specific hits in alpha-factor arrested cells, S-phase (HU) and late anaphase (*cdc15-1*). **(B)** Summary and overlap of the candidate proteins selected for further validation.

Taken together, 88 specific interaction partners of the septins (including the five mitotic septins) were identified (Fig 4A). Only nine proteins (including the five mitotic septins) were present in all three stages (Fig 4A) and each four common interaction partners were identified for alpha factor arrest and S phase and for S phase and anaphase, respectively. None of the identified proteins bound to the septins both in alpha factor arrest and late anaphase.

Representative candidates were randomly picked from the different functional categories for validation by complementary methods. A total of 26 proteins were selected for validation experiments (Fig 4B).

### A variation of the SPLIFF system provides a fast and robust validation platform for MS based interaction hits

The interactions of selected candidates with the septins were analyzed *in vivo* using a variation of the SPLIFF technique. SPLIFF is a method for the detection of protein-protein interactions in a time- and spatial resolved manner inside a living cell [21,26]. It is based on the split-ubiquitin method [34] and uses two spectrally different fluorophores as interaction signal. A mCherry-C_ub_-GFP (CCG) module is attached to the C-terminus of a protein of interest, whereas N_ub_ is fused to the N-terminus of a potential interaction partner. An interaction between the two proteins induces a reassociation of the ubiquitin halves N_ub_ and C_ub_. This native-like ubiquitin is then recognized by a ubiquitin-specific protease that cleaves off the GFP from the C_ub_. The liberated GFP carries a genetically inserted N-terminal destabilizing amino acid and is rapidly degraded through the ubiquitin-proteasome pathway. The ratio of GFP to mCherry fluorescence indicates the extent of interaction. After examining the effects of tagging all mitotic septins with the CCG module on cell growth and morphology (S4 Fig), we decided to use Shs1-CCG as least disturbing CCG fusion to the septin rod. The acceptor of the label thus differs between the SPLIFF and the AP-MS experiments. We established a “steady-state” SPLIFF as a fast screen for interaction partners of the septins *in vivo*. A Shs1-CCG strain was mated with strains of the opposite mating type expressing different candidate N_ub_-fusions under control of the copper-inducible *P*_*CUP1*_-promoter. Resulting diploid cells were grown in the presence and absence of Cu^2+^ to exponential phase and the interaction was quantified by determining the ratios of GFP over mCherry fluorescence at the bud neck of individual cells. Thresholds for a strong and weak interaction were set to a CCG to CC conversion (SPLIFF signal) of greater than 70% and greater than 30%, respectively. SPLIFF signals lower than 30% were assigned as non-interacting. See the Supplementary Methods (S1 Methods) section for the calculation of the SPLIFF signal and the S2 Table for the corresponding values.

**Fig 5:**
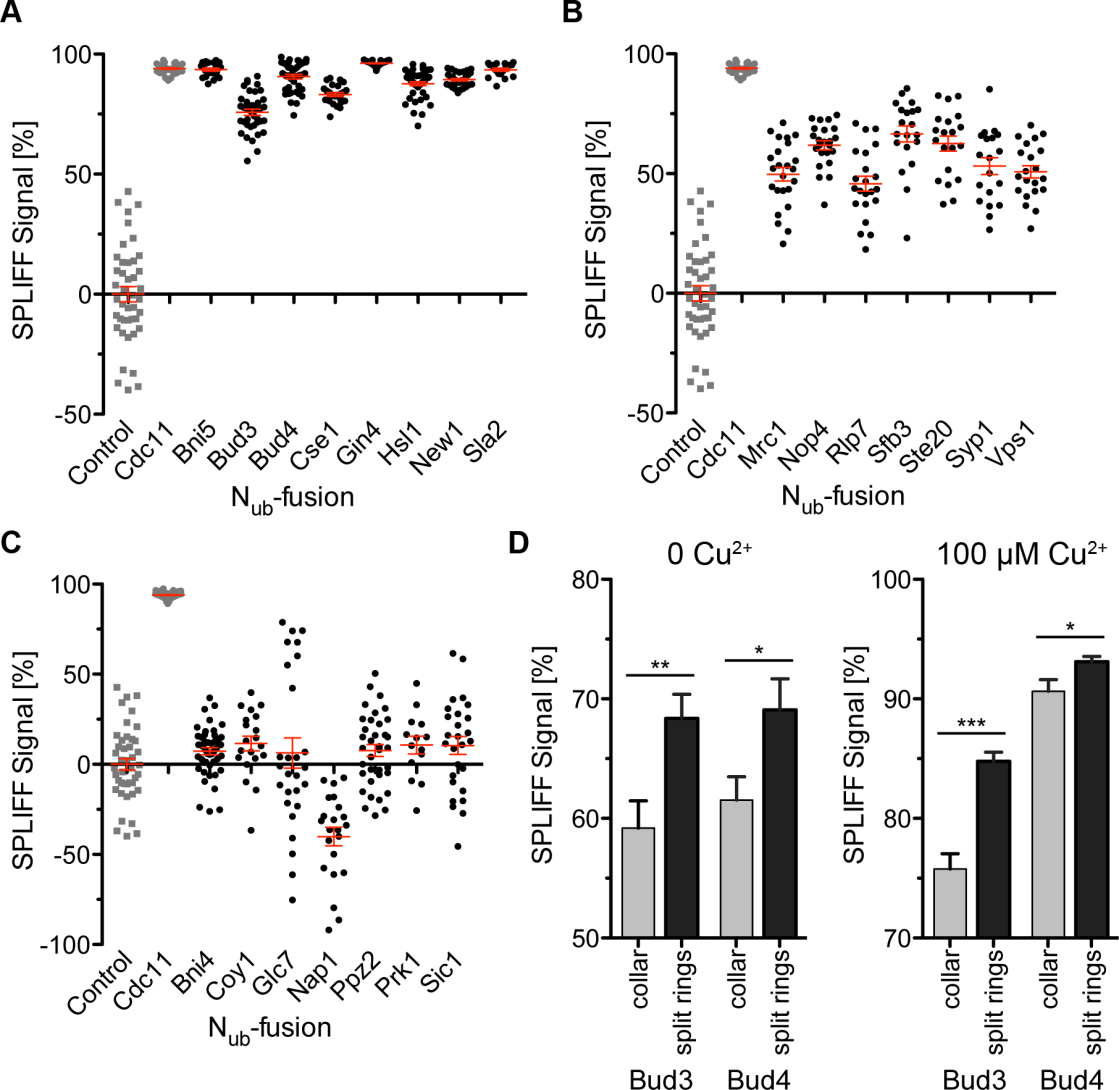
Steady-state SPLIFF measurements. Each point represents a single cell measurement. The expressions of the N_ub_ -fusion proteins were induced by100 μM Cu^2+^. The calculated medians and SEMs are shown in red. N_ub_-fusions are grouped according to their interaction signal. (**A**) Strong interaction (SPLIFF signal ≥ 70%). (**B**) Weak interaction (SPLIFF signal ≥ 30%). (**C)** No interaction (SPLIFF signal ≤ 30%). The negative value for Nap1 might result from a weaker expression of Nap1 than the control N_ub_. Note a subpopulation of Glc7 cells that have a SPLIFF signal >50%. grey squares: negative control N_ub_-empty; grey circle: positive control N_ub_-Cdc11. (**D**) Comparison between stable collar and split septin rings SPLIFF signals induced by N_ub_- and -Bud4 with 0 (left) or 100 μM Cu^2+^ (right).

Under *P*_*CUP1*_-inducing conditions of the 26 examined candidate proteins, eight proteins were classified as strong and seven as weak interactors. Seven proteins were assigned as non-interacting with the septins based on the SPLIFF analysis (Fig 5A–5C). Four candidate proteins could not be labeled with N_ub_. The results of all SPLIFF interaction assays are summarized in Table 1. The result of the SPLIFF analysis for all evaluated candidates under non-inducing conditions is shown in the S5 Fig.

**Table 1:** SPLIFF- and alternative evaluation of 26 selected candidates.

Candidate protein	Category	MS identification point	SPLIFF validation (in vivo interaction with septins)	Pulldown validation (in vitro interaction with septins)	Annotations	Figure reference
Bni5	scaffold	α-F, S-phase, anaphase	strong interaction	see [36]	Known septin interactor [37,38]	3A-C, 5A
Glc7	regulatory	α-F, S-phase, anaphase	no interaction			3A-C, 5C
Bni4	regulatory	α-F, S-phase, anaphase	no interaction		Time lapse microscopy: colocalization with septins. Known septin interactor [35]	3A-C, 5C, 6A
Ppz2	regulatory (phosphatase)	α-F, S-phase, anaphase	no interaction			3A-C, 5C
New1	ribosome/protein synthesis	α-F, S-phase	strong interaction	n/a **		3A, 3B, 5A, S7
Nip1	ribosome/protein synthesis	α-F, S-phase	n/a *	n/a **	Colocalization in arrested cells: no interaction	3A, 3B, 6B
Cse1	DNA interacting/nucleus	α-F	strong interaction			3A, 5A
Nop4	DNA interacting/nucleus	α-F	weak interaction			3A, 5B
Rlp7	DNA interacting/nucleus	α-F	weak interaction	negative		3A, 5B, S7
Pno1	DNA interacting/nucleus	α-F	n/a *	positive	Colocalization in arrested cells: no interaction	3A, 6B, S7
Sfb3	protein sorting/endocytosis	α-F	weak interaction	positive		3A,5B, S7
Vps1	protein sorting/endocytosis	α-F	weak interaction	positive		3A, 5B, S7
Sla2	protein sorting/endocytosis	S-phase	strong interaction	positive		3B, 5A, S7
Coy1	protein sorting/endocytosis	S-phase	no interaction			3B, 5C
Syp1	protein sorting/endocytosis	anaphase	weak interaction	positive	Known septin interactor [32]	3C, 5B, S7
Gin4	regulatory (kinase)	S-phase, anaphase	strong interaction		Known septin interactor [15]	3B, 3C, 5A
Nap1	regulatory	S-phase, anaphase	no interaction			3B, 3C, 5C
Prk1	regulatory (kinase)	S-phase	no interaction			3B, 5C
Hsl1	regulatory (kinase)	S-phase	strong interaction		Known septin interactor [39]	3B, 5A
Ste20	regulatory (kinase)	anaphase	weak interaction	positive	Known septin interactor [40]	3C, 5B, S7
Sic1	cell cycle control	anaphase	no interaction			3C, 5C
Mrc1	cell cycle control	anaphase	weak interaction			3C, 5B
Smt3	regulatory	anaphase	n/a *		SPLIFF with expression from plasmid. Known interactor [16]	3C, S4
Tub1	scaffold	anaphase	n/a *	n/a **	Colocalization in arrested cells: no interaction	3C, 6B
Bud4	regulatory	S-phase, anaphase	strong interaction	positive	Known septin interactor [41]	3B, 3C, 5A, 5D, 7C
Bud3	scaffold	anaphase	strong interaction	positive	Known septin interactor [42]	3C, 5A, 5D, 7C

(*) a N_ub_ fusion could not be created

(**) proteins bound unspecifically

**Fig 6:**
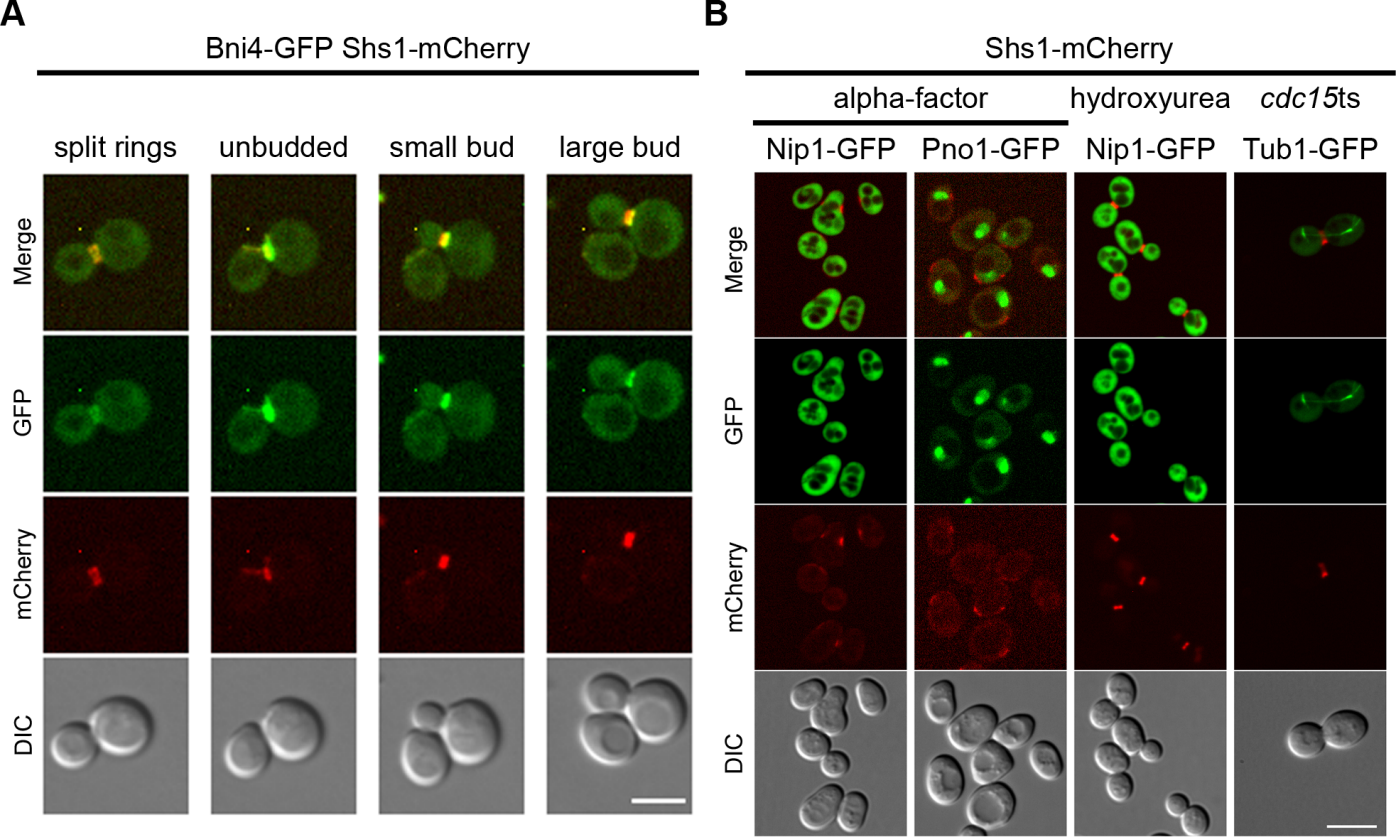
Validation of selected candidate proteins by fluorescence microscopy. (**A**) Live cell imaging of cells expressing Shs1-mCherry and Bni4-GFP at indicated time points of the cell cycle. Scale bar 5 μm. (**B**) Fluorescence microscopy images of blocked cells expressing Shs1-mCherry and Nip1-, Pno1-or Tub1-GFP. Scale bar 6 μm.

The SPLIFF analysis classified Bni4 as non-interactor, although the protein had been previously described as septin interacting [35,36]. We could confirm that Bni4-GFP co-localizes with the septins throughout the cell cycle (Fig 6A). Co-localization studies were also performed with three of those proteins (Nip1, Pno1 and Tub1) that did not tolerate the N-terminal N_ub_-fusion. In none of these cases we observed co-localization with the septins (Fig 6B). The fourth protein, Smt3, represents a known septin interactor [16]. We performed steady-state SPLIFF with N_ub_-Smt3 expressed from a centromeric plasmid in this case. We observed a mixed population of cells with an enlarged phenotype showing the interaction and of cells with normal size without interaction signal (S6 Fig).

To substantiate the finding of our SPLIFF analysis, we expressed a random selection of candidates that were positively evaluated in the SPLIFF analysis as TAP- or GFP tagged proteins in *S*. *cerevisiae* and performed a pulldown on purified septin rods. We tested also the proteins that did not tolerate the N_ub_-fusion (Nip1, Pno1, Tub1). Clear signals above background validated Sfb3, Sla2, Ste20, Syp1, Pno1 and Vps1 as specific binding partners (S7A Fig).

The extent of unspecific binding to the matrix excluded New1, Nip1 and Tub1 (S7B Fig, data not shown for the latter two) from the analysis.

Rlp7 showed a weak interaction in the SPLIFF analysis but did not bind to the septin rods in the pulldown analysis (S7C Fig). Bni5 was previously evaluated with the same method as septin interacting protein [37]. All pulldown results are summarized in Table 1 and shown in S7 Fig.

### Spatial and temporal refinement of MS defined interactions: The interaction between Bud3 and Bud4 and the septins as a test case

**Fig 7:**
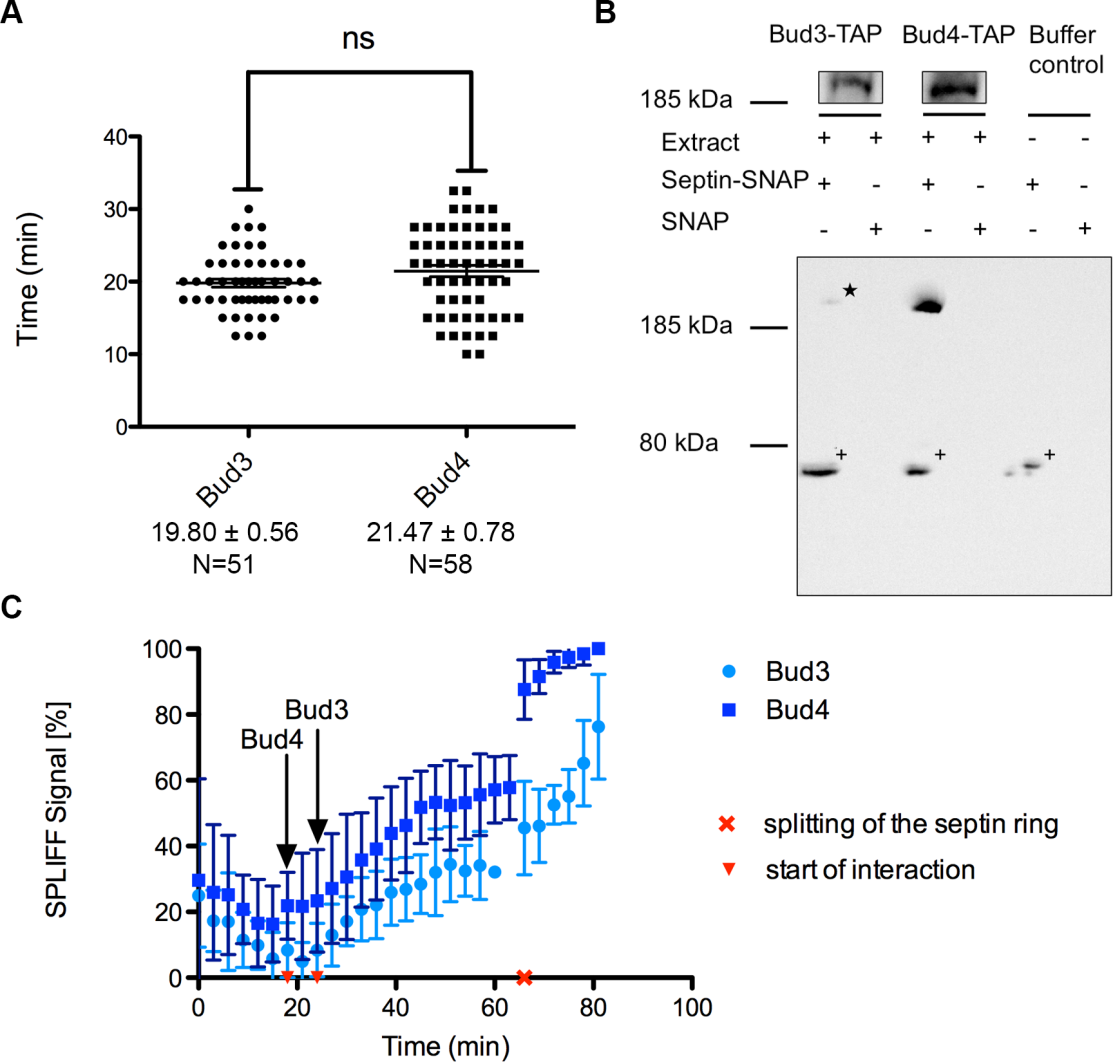
Timing of the interaction of Bud3 and Bud4 with the septins. **(A)** Time lapse microscopy of Bud3-GFP or Bud4-GFP and Shs1-mCherry expressing cells. The time of the first appearance of the GFP fusion protein after the appearance of Shs1-mCherry at the bud neck was recorded. **(B)** Pulldown of Bud3-TAP and Bud4-TAP on immobilized septin rods and detection with an anti-Protein A antibody. The asterisk marks the hardly visible Bud3-TAP signal. A cross reaction of the antibody with one of the septins is marked with +. **(C)** Time resolved SPLIFF analysis (N_ub_-Bud3 or N_ub_-Bud4 vs. Shs1-CCG). Imaging time frame 3 min, 100 μM Cu^2+^. N = 10 for Bud3 and N = 9 for Bud4. The start of the N_ub_ induced conversion of Shs1-CCG is marked with an arrow.

Bud3 and Bud4 are known to interact with each other and with the septins in an interdependent manner [43,44]. They both co-localize at the bud neck well before the onset of mitosis. In agreement with published data [42] we could localize the GFP fusions of both proteins in Shs1-mCherry co-expressing cells to the incipient bud site approximately 20 minutes after the septin patches became detectable at this site (Fig 7A). From this time point on the distributions of both fusions closely match the cellular localization of the septins including the splitting into a double ring during cytokinesis.

In contrast to the strict co-localization, our MS screen identified Bud4 in S-phase and late anaphase as septin interactor whereas Bud3 was unexpectedly found to be associated with the septins only at mitosis during the splitting of the septin rings (Fig 4). Bud3 and Bud4 were both validated as strong interaction partners of the septins in the steady-state SPLIFF analysis (Fig 5A). We noticed that the SPLIFF signal of N_ub_-Bud3 and N_ub_-Bud4 further increases in cells with split septin rings in comparison to cells with a stable septin collar (Fig 5D).

To resolve the discrepancy between co-localization- and protein interaction data we followed the interaction of Bud3 and Bud4 with the septins during the 90 min of a single cell cycle by time resolved SPLIFF analysis using N_ub_-Bud3 or N_ub_-Bud4 and Shs1-CCG as the corresponding Split-Ub fusion proteins (Fig 7B).

The interaction between N_ub_-Bud4 and Shs1-CCG started shortly after the fusion of the N_ub_-Bud4 and Shs1-CCG expressing yeast cells. The linear conversion of Shs1-CCG lasted till min 45 and paused for 18 min before abruptly continuing during the splitting of the septin rings. The kinetics of the N_ub_-Bud3 induced conversion of Shs1-CCG was very similar except that initiation and the linear increase were shifted by more than 6 min into the cell cycle. As observed for N_ub_-Bud4, the N_ub_-Bud3-induced conversion of Shs1-CCG stopped at min 45. We conclude that Bud4 interacts with the septins immediately upon arrival at the incipient bud site whereas Bud3 is initially recruited by Bud4 and only later able to directly interact with the septins.

We finally asked whether Bud3 and Bud4 are both direct interaction partners of the septins. We purified SNAP tagged septin rods and immobilized them on BG-Sepharose beads. The beads were incubated with extracts from yeast expressing Bud3-TAP or Bud4-TAP. Bud4 was specifically eluted from septin-coated beads whereas Bud3 did only show a weak interaction (Fig 7C).

## Discussion

To determine septin-associated interactomes in dependence of the cell cycle, we blocked the cell cycle at three distinct stages of septin architecture: i) in alpha-factor arrested cells (structure before ring assembly), ii) in S-phase (stable septin collar; block by HU), and iii) in late anaphase (split septin rings; *cdc15-1* temperature-sensitive mutant).

The *cdc15-1*-allele has been described to arrest the cell cycle in late anaphase [28], before the splitting of the septin collar. However, we found that *cdc15-1*-arrested cells displayed split septin rings to a high percentage of about 86%. The reduction of the temperature during the visualization- or sample preparation process probably led to a partial release from the arrest and subsequently promoted the splitting of the “instable” septin collar. The associated interaction partners might have partially accompanied this change in septin structure, and the identified interactome might represent more the split septin architecture than the mitotic septin ring. However, the situation is not as clear-cut as described as we find Bni5 as interaction partner of the split septin ring in *cdc15-1* cells whereas Bni5 was shown in wild type cells to leave the septin ring cells shortly before splitting [37,38].

Using Cdc11-TAP as target, our quantitative MS analyses revealed 83 potential new physical interaction partners of Cdc11. It is however important to realize that not Cdc11 alone but the intact rod forms the bait during the affinity precipitations. All found interaction partners are therefore binders of the septin rod. To reveal the identity of the septin subunit that interacts with a certain partner protein would require additional experiments that exceed the scope of this work.

The interaction of 26 selected candidate proteins was further validated *in vivo* by steady-state SPLIFF measurements. We chose SPLIFF as a truly orthogonal validation technique and deliberately refrained from the commonly used IPs. While IPs represent a well established *in vitro* validation method, SPLIFF independently measures interactions in their native environment inside the living cell.

SPLIFF, in contrast to the standard Split-Ubiquitin assay, allows the detection of weak interactions because no complete degradation of the C_ub_-attached reporter is required to obtain an interaction signal [21]. The standard SPLIFF method requires the time-resolved measurement of the interaction and is thereby not suitable for screening a large number of interaction partners. To circumvent this limitation, we established the steady-state SPLIFF. Diploid cells expressing Shs1-CCG and a N_ub_-candidate fusion protein were grown to exponential phase and subjected directly to fluorescence microscopy. This method benefits from the feature of the septins to form a stable structure at the bud neck during nearly the complete cell cycle that comprises the inherited as well as the newly synthesized Shs1-CCG molecules [45].

We used Shs1 as attachment site for the CCG module as in contrast to the other tested septin subunits, Shs1-CCG looked indistinguishable from the GFP-labeled septins during the cell cycle. However, by restricting the SPLIFF substrate to the terminal Shs1 we had to keep in mind that a) the three-dimensional structure of Shs1 containing septins might differ from the one of Cdc11 containing septins [11] and b) that the analysis will sample only those interactors that locate close the terminal subunits of the septin rods. Proteins that bind to the center of the octamers or exclusively to Cdc11 might thus not be detected. Without knowledge of the nearest subunit neighbors of Shs1 in the different higher-order septin structures it is difficult to discuss why a certain interaction revealed by TAP precipitation is not seen by SPLIFF. Bni4 is a relevant example. This protein comes up as interaction partner in the AP-MS analysis during all three stages but is not revealed by SPLIFF as interaction partners of the septins. Bni4 was shown by Two-Hybrid analysis to interact only with Cdc10 [35,36], the central subunit of the septin rod. The central location of the coupled N_ub_ might thus prevent the generation of a SPLIFF signal with a peripherically located Shs1-CCG. Again, repeating the SPLIFF analysis with all other septin subunits as CCG-fusions could eliminate some of the encountered discrepancies. However, the majority of proteins that were evaluated as positive interactors in the pulldown analysis were also found as positive interactors in the SPLIFF analysis.

In particular the prevalence of nuclear proteins and proteins involved in ribosome biogenesis among the septin interactors is puzzling as the septins were never reported to stay in the nucleus. In alpha-factor treated cells, proteins of these two categories represent the majority of the identified interactors (together 23 proteins, 57.5%). Of these proteins, 20 are described to localize to the nucleus or nucleolus [46] with the majority (17) being involved in ribosomal RNA biosynthesis and processing or biogenesis of ribosomal subunits as annotated in the Saccharomyces genome data base (SGD). Before flatly rejecting these hits as artefacts of abundant and perhaps sticky proteins one has to consider three arguments that support the specificity of the measured interactions. 1. Most of those hits are only reported for alpha-factor-treated cells and not for the other two cell cycle stages. 2. Three proteins involved in ribosomal RNA processing (Nop4, New1 and Rlp7) and one nuclear protein, Cse1, were validated as septin interactors by SPLIFF analysis. 3. The applied SILAC approach is especially good in avoiding these kind of artefacts. Independent of these considerations, the potential cellular significance of these interactions remains enigmatic.

Three endocytic proteins were found to interact with the septins at the three different time points: Vps1 in early alpha factor arrested cells, Sla2 in S-phase and Syp1 in late anaphase.

Of these only Syp1, which is involved in endocytic site formation, was already shown to interact with the septins [32,47]. Furthermore, a role of Syp1 in maintaining septin ring stability has been suggested [48]. The mechanism proposed by Merlini et al. opens the possibility that Syp1 is involved in the coordination of membrane remodeling controlled by mechanical stress at the septin ring.

Overall, the identification of endocytic proteins as septin interactors supports the assumption that septins foster endocytosis and bias the polarized distribution of cargo material in the plasma membrane [49].

The septins are known targets of cell cycle regulated post-translational modifications. Phosphorylations, SUMOylations and N-acetylations of the septins are involved in the transitions of the septin structure during the cell cycle [10]. Our quantitative MS-analysis revealed distinct sets of regulatory proteins that might perform these modifications at different stages of the cell cycle. In particular, the set of regulatory proteins in cells with a stable collar varies considerably from the set of proteins associated with split rings. The kinase Gin4 and the anillin-like protein Bud4 were identified in both time points. Three kinases, Hsl1, Mck1 and Prk1 are specifically associated with the septins in cells with a stable collar. Another kinase (Ste20) and yeast SUMO (Smt3) were only identified as specific septin interactors in cells with split septin rings. The septins Cdc3, Cdc11 and Shs1 are known to be SUMOylated at the G_2_/M transition and de-SUMOylated shortly before cytokinesis [16]. This timing agrees well with Smt3 being precipitated with the septins only from the extracts of the *cdc15-1* arrested cells.

For three of the five identified kinases (Gin4, Hsl1 and Ste20), a physical interaction with the septins is described in literature [39,40,50,51]. Gin4 is recruited together with the septins to the presumptive bud site, co-localizes with the septins at the bud neck for the complete cell cycle and disappears from the bud neck after splitting of the septin collar [52]. This timing is in good agreement with our MS analysis. The Gin4 homologue in *C*. *albicans* was recently shown to regulate Nap1 by phosphorylation, a protein being directly involved in septin organization [53]. Gin4 and Nap1 were both identified in this study as interaction partners of the septins in cells with a stable septin collar and split rings. Our data thus support the assumption that Gin4 possesses the same functions in *S*. *cerevisiae* as discovered in *C*. *albicans*.

Recently, it was shown that Bud4 possesses a crucial role in the assembly of the axial landmark. Bud4 is recruited to the bud neck via an association with the septins and builds up a platform for the assembly of the other components of the axial landmark [41,54].

Bud3 and Bud4 were thought to interdependently and simultaneously bind to the septins after disassembly from this landmark. However, our MS study and subsequent steady state SPLIFF analysis suggested that Bud3 binds to the septins later than Bud4 although both proteins always localize together at the bud neck.

We used a time and spatially resolved SPLIFF analysis to explain this discrepancy. We could show that Bud4 indeed interacts with the septins at least 6 min earlier than Bud3. After this lag time both N_ub_-fusions convert Shs1-CCG with the same kinetics and cease to interact with Shs1-CCG at exactly the same time. During the splitting of the septins the interactions suddenly resume.

Without further knowledge about the nature of the interactions between the septins and Bud4 and Bud3 these striking kinetic patterns are difficult to explain. However, a simple model can account for this pattern if one assumes that the interaction between the septins and Bud3 and Bud4 are supported by two different post-translational modifications of the septins. The model would than postulate that the first modification precedes and perhaps is required for the second modification and that Bud4 binds preferentially to septins harbouring the first and Bud3 to septins harbouring the second modification.

## Supporting Information

S1 Fig Incorporation of “heavy” arginine and lysine. Saturated overnight cultures grown in “heavy” SILAC medium were diluted into fresh “heavy” SILAC medium. Cells were incubated at 30°C for 7.5 h and the incorporation of “heavy” amino acids was analyzed by MS. The percentage of all measured peptide pairs was plotted against the percentage of incorporation.
(TIF)

S2 Fig Affinity purifications of arrested cells. Representative overviews of affinity purifications of Cdc11-fusions from extracts of α-factor- (I), HU- (II) or *cdc15-1*-arrested cells (III). From left to right: anti-Protein A Western Blots of the purification of Cdc11-TAP (arrowhead: TEV-protease cleaved Protein A), anti-GFP Western Blots of the control Cdc11-GFP, colloidal Coomassie stains of the eluates. The bands were subsequently excised and used for quantitative MS-analyses. Ex: extract, S: supernatant, P: pellet, FT: flow-through, W: wash, E: Elution with TEV-Protease, BB: boiled beads. MAP: "mixing after purification".
(TIF)

S3 Fig Overlap of the identified proteins. Venn diagrams showing the overlap of all identified proteins in the three replicates of (A) G1-phase (alpha factor), (B) S1-phase, (C) anaphase. Specific interactors were grouped into the indicated categories. Percentages of specific interactors for each category are presented.
(TIF)

S4 Fig Tagging of Septin subunits with the CCG module. Fluorescence microscopy of cells expressing Cdc3-CCG, Cdc10-CCG, Cdc11-CCG, Cdc12-CCG and Shs1-CCG. Only cells expressing Shs1-CCG do not display a visible phenotype. Scale bar 5 **μ**m.
(TIF)

S5 Fig Steady-state SPLIFF measurements with 0 μM Cu^2+^ (restrictive conditions). Each point represents a single cell measurement. The calculated medians and SEMs are shown in red. N_ub_-fusions are grouped according to their interaction signal. (**A**) Strong interaction (SPLIFF signal ≥ 70%). (**B**) Weak interaction (SPLIFF signal ≥ 30%). (**C**) No interaction (SPLIFF signal ≤ 30%). grey squares: negative control N_ub_-empty; grey circle: positive control N_ub_-Cdc11.
(TIF)

S6 Fig Phenotypes of diploid cells expressing Shs1-CCG and N_ub_-Smt3. Fluorescence microscopy images of diploid cells expressing Shs1-CCG and N_ub_-Smt3 (from plasmid) at 0 μM Cu^2+^ (upper panel) and 100 μM Cu^2+^. Scale bar 3 μm.
(TIF)

S7 Fig Pulldown analysis of selected candidate proteins. **(A)** Beads covalently captured with recombinant septin rods or SNAP tag were incubated with extracts from yeast expressing the indicated TAP tagged (Vps1: GFP-tagged) candidate proteins. After washing, specific binders were eluted in Lämmli buffer and monitored via an anti-protein A or anti-GFP antibody. Left lanes: immobilized SNAP tagged septin rods. Right lanes: SNAP tag protein (control). The band at approx. 70 kD results from a cross reaction of the primary antibody with one of the septins. **(B)** Unspecific binding of New1 to the pulldown matrix. **(C)** Rlp1 does not bind to immobilized septin rods (left). The loading control including extract (Ex) and Pellet (P) indicates soluble expressio (right). **(D)** Representative input control: the 6his-tagged septin rod subunit Cdc12 and 6his-SNAP were detected with an anti-his antibody. For each candidate a representative blot out of minimum two independent replicates is shown. The asterisk marks the expected protein size.
(TIF)

S1 Methods (PDF)

S1 Table Proteins identified in AP-MS analyses of Cdc11-complexes purified from cells arrested woth alpha factor (G1) **(A)**, S1 phase **(B)** and late anaphase **(C).**
(XLSX)

S2 Table Steady-state SPLIFF analysis data. (PDF)

S3 Table Yeast strains used in this work. (PDF)
