# Supplementary figures and images for: Identification of Cell Cycle Dependent Interaction Partners of the Septins by Quantitative Mass Spectrometry
Source: PLoS One. 2016 Feb 12;11(2):e0148340. doi: 10.1371/journal.pone.0148340 (PMC4752459; doi:10.1371/journal.pone.0148340)

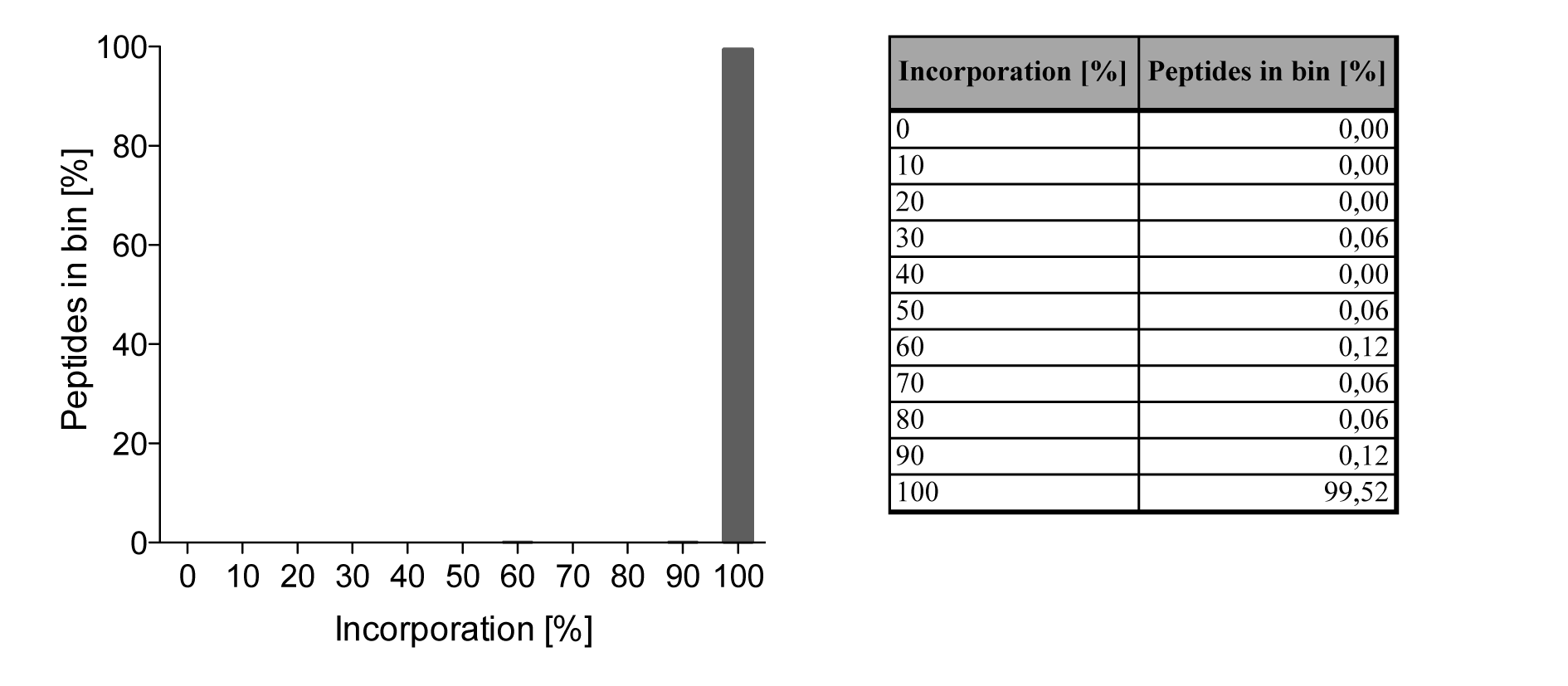

Supplement: S1 Fig — Saturated overnight cultures grown in “heavy” SILAC medium were diluted into fresh “heavy” SILAC medium. Cells were incubated at 30°C for 7.5 h and the incorporation of “heavy” amino acids was analyzed by MS. The percentage of all measured peptide pairs was plotted against the percentage of incorporation. (TIF) [file pone.0148340.s001.tif]

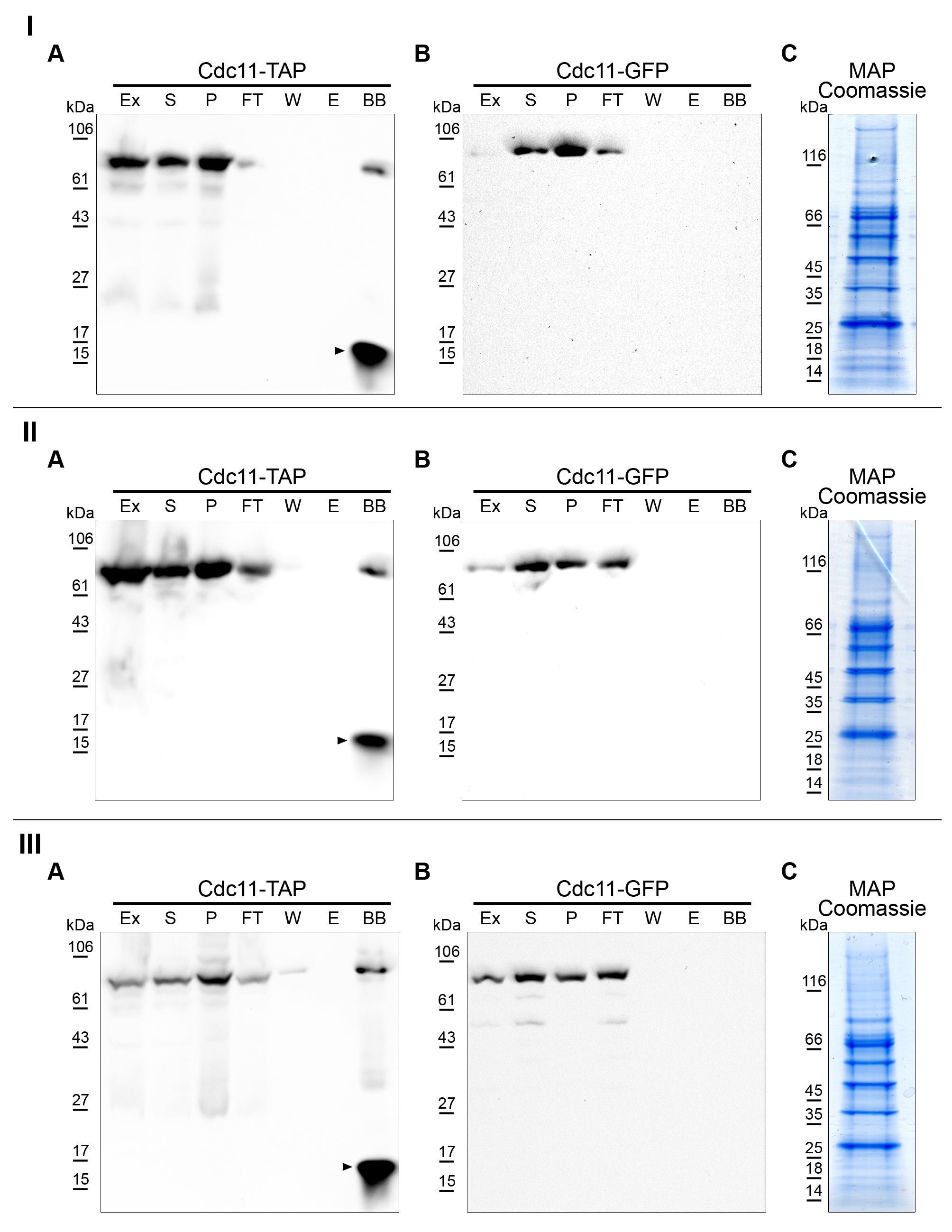

Supplement: S2 Fig — Representative overviews of affinity purifications of Cdc11-fusions from extracts of α-factor- (I), HU- (II) or cdc15-1-arrested cells (III). From left to right: anti-Protein A Western Blots of the purification of Cdc11-TAP (arrowhead: TEV-protease cleaved Protein A), anti-GFP Western Blots of the control Cdc11-GFP, colloidal Coomassie stains of the eluates. The bands were subsequently excised and used for quantitative MS-analyses. Ex: extract, S: supernatant, P: pellet, FT: flow-through, W: wash, E: Elution with TEV-Protease, BB: boiled beads. MAP: "mixing after purification". (TIF) [file pone.0148340.s002.tif]

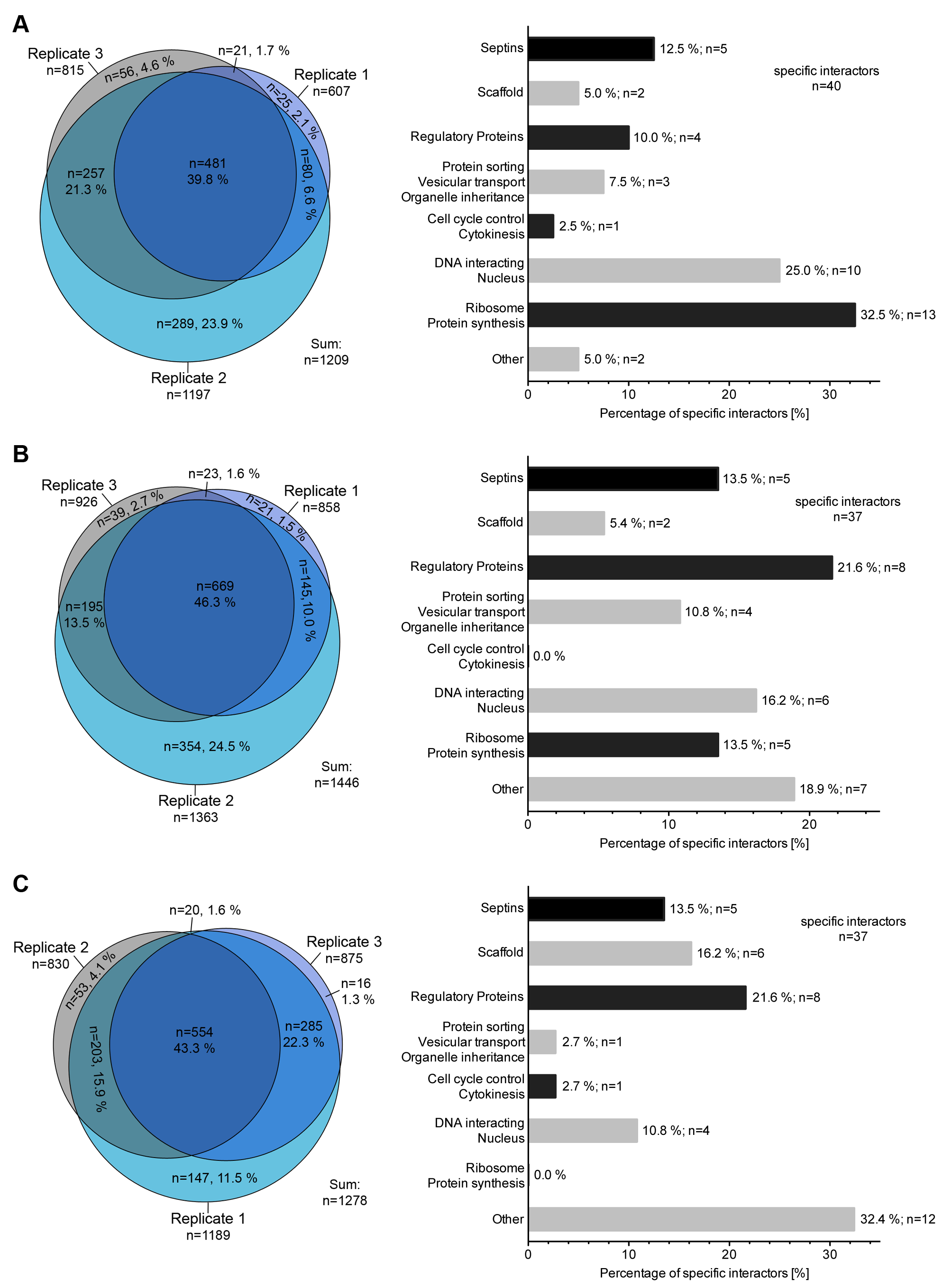

Supplement: S3 Fig — Venn diagrams showing the overlap of all identified proteins in the three replicates of (A) G1-phase (alpha factor), (B) S1-phase, (C) anaphase. Specific interactors were grouped into the indicated categories. Percentages of specific interactors for each category are presented. (TIF) [file pone.0148340.s003.tif]

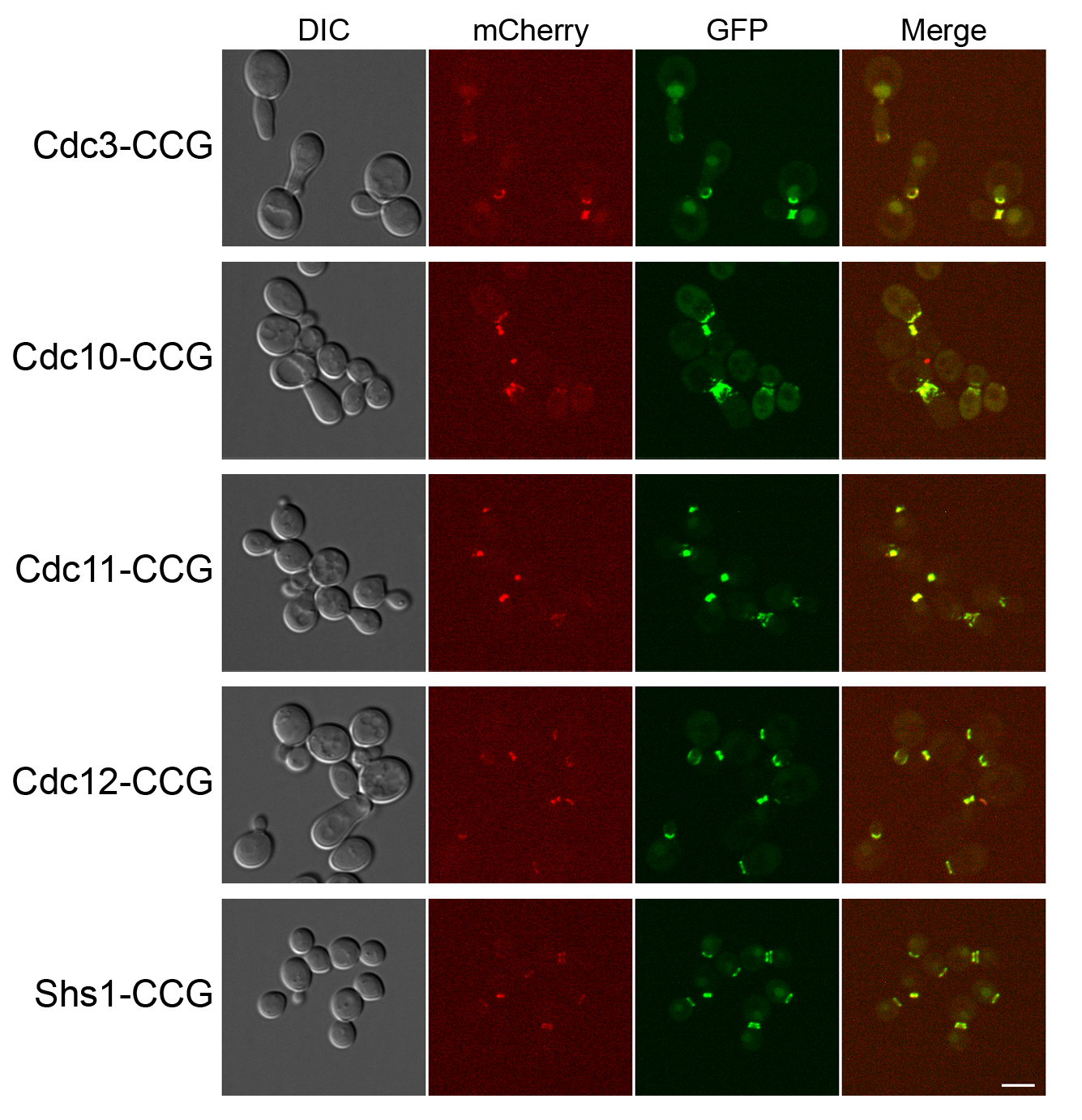

Supplement: S4 Fig — Fluorescence microscopy of cells expressing Cdc3-CCG, Cdc10-CCG, Cdc11-CCG, Cdc12-CCG and Shs1-CCG. Only cells expressing Shs1-CCG do not display a visible phenotype. Scale bar 5 μm. (TIF) [file pone.0148340.s004.tif]

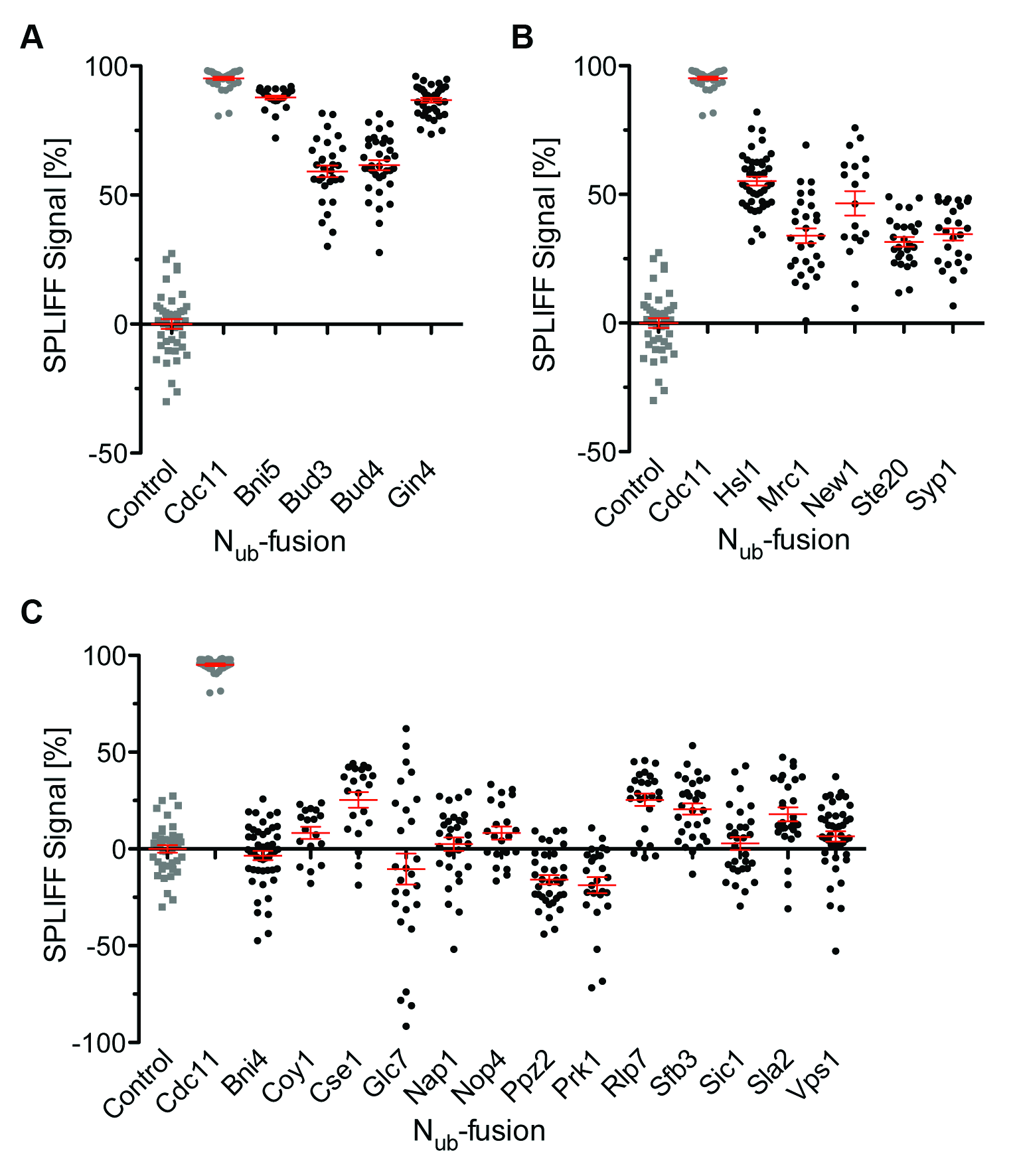

Supplement: S5 Fig — Each point represents a single cell measurement. The calculated medians and SEMs are shown in red. Nub-fusions are grouped according to their interaction signal. (A) Strong interaction (SPLIFF signal ≥ 70%). (B) Weak interaction (SPLIFF signal ≥ 30%). (C) No interaction (SPLIFF signal ≤ 30%). grey squares: negative control Nub-empty; grey circle: positive control Nub-Cdc11. (TIF) [file pone.0148340.s005.tif]

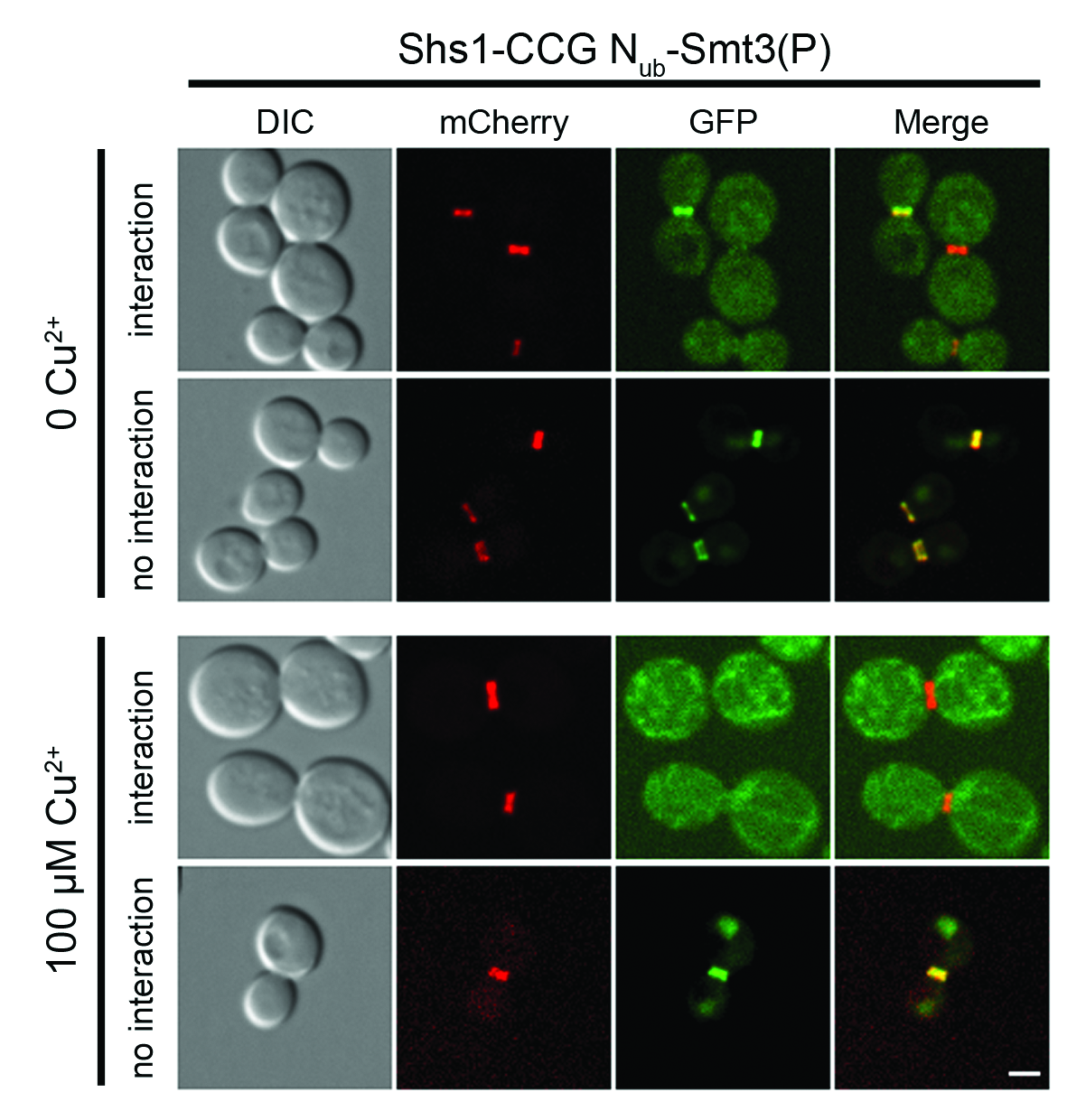

Supplement: S6 Fig — Fluorescence microscopy images of diploid cells expressing Shs1-CCG and Nub-Smt3 (from plasmid) at 0 μM Cu2+ (upper panel) and 100 μM Cu2+. Scale bar 3 μm. (TIF) [file pone.0148340.s006.tif]

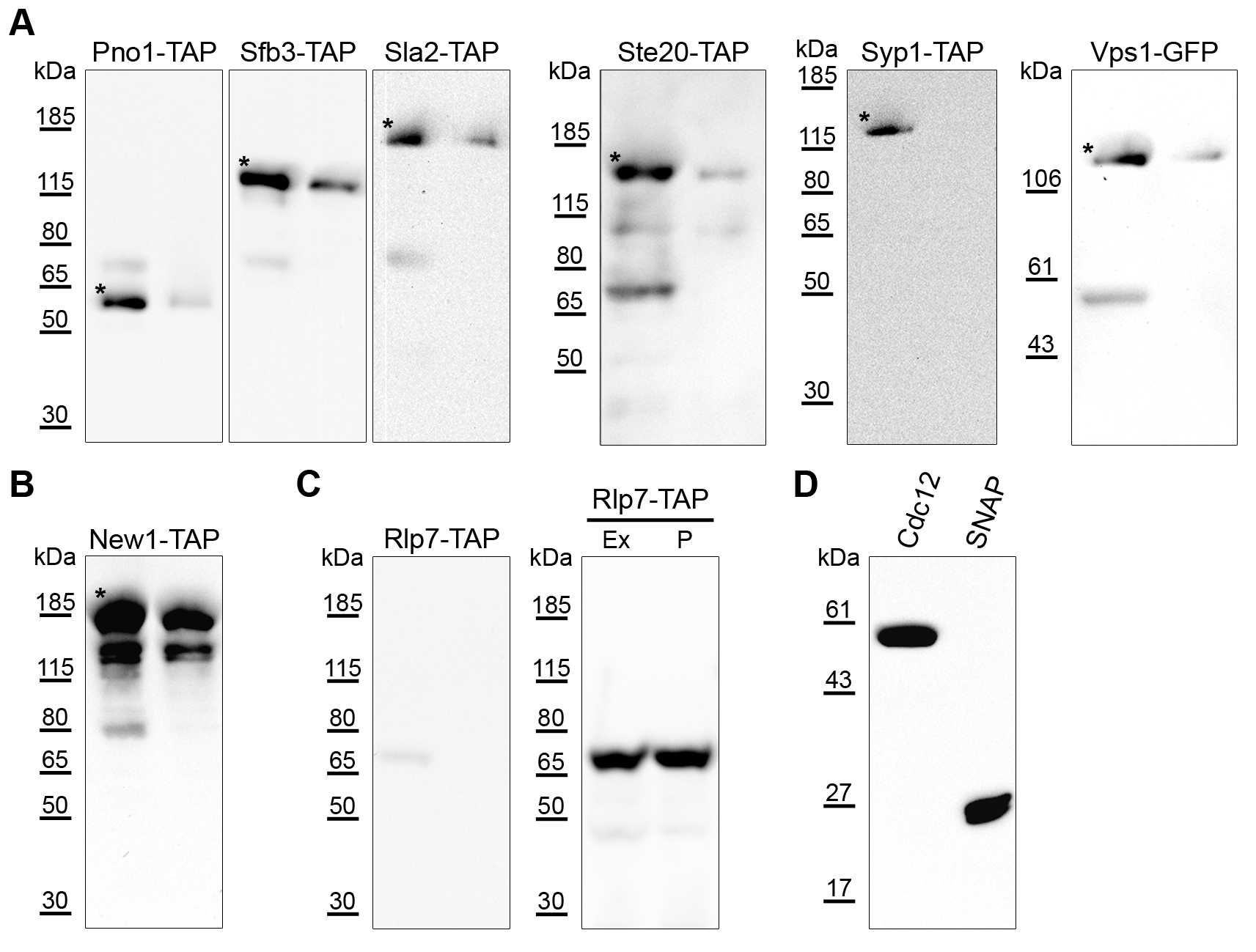

Supplement: S7 Fig — (A) Beads covalently captured with recombinant septin rods or SNAP tag were incubated with extracts from yeast expressing the indicated TAP tagged (Vps1: GFP-tagged) candidate proteins. After washing, specific binders were eluted in Lämmli buffer and monitored via an anti-protein A or anti-GFP antibody. Left lanes: immobilized SNAP tagged septin rods. Right lanes: SNAP tag protein (control). The band at approx. 70 kD results from a cross reaction of the primary antibody with one of the septins. (B) Unspecific binding of New1 to the pulldown matrix. (C) Rlp1 does not bind to immobilized septin rods (left). The loading control including extract (Ex) and Pellet (P) indicates soluble expressio (right). (D) Representative input control: the 6his-tagged septin rod subunit Cdc12 and 6his-SNAP were detected with an anti-his antibody. For each candidate a representative blot out of minimum two independent replicates is shown. The asterisk marks the expected protein size. (TIF) [file pone.0148340.s007.tif]
