# Supplementary material for: Identification of Cell Cycle Dependent Interaction Partners of the Septins by Quantitative Mass Spectrometry
Source: PLoS One. 2016 Feb 12;11(2):e0148340. doi: 10.1371/journal.pone.0148340 (PMC4752459; doi:10.1371/journal.pone.0148340)
Supplement: S1 Methods — (PDF) [file pone.0148340.s008.pdf]

Supplementary Methods

Construction of the yeast strains

For SILAC-based protein expression, a BY4142 based strain (*MAT*a *arg4::KanMX4 his3Δ1 leu2Δ0 lys1::KanMX4 lys2Δ0 ura3Δ0*) was used (kindly provided by M. Perutz Laboratories, Vienna, A). The *TRP1* locus of this strain was replaced by *HIS3* yielding BY-SILAC.

For the generation of the SILAC control strain, the 3' end of *CDC11* was cloned in frame with GFP into pRS304 (Sikorski & Hieter, 1989). The obtained plasmid was linearized using a unique restriction side in *CDC11* and integrated in BY-SILAC using the *TRP1* marker of the plasmid. The SILAC Cdc11-TAP strain was created by PCR-based one step integration of the TAP-tag including a *TRP1* locus at the Cdc11 C-terminus as described previously (Rigault et al, 1999).

A BY-SILAC strain carrying the temperature sensitive *cdc15-1* allele (bearing a single point mutation, A206D) was constructed as follows: a *LEU2* marker cassette (PCR-amplified from plasmid pFA6a-C.m.*LEU2* (Euroscarf)) was integrated 300 bp downstream of the *CDC15* locus in the strain ESM1278 carrying the *cdc15-1* allele (Pereira et al, 2002). The *cdc15-1* allele including the *LEU2* marker cassette was PCR-amplified from the modified strain and integrated at the *CDC15* locus of the corresponding BY-SILAC strain using the *LEU2* marker cassette. The resulting clones were screened for temperature sensitivity at 37°C and the integrity of the *cdc15-1* allele was verified by sequencing. *CDC11*-GFP and *CDC11*-TAP were integrated into this strain as described above.

For fluorescence microscopy, GFP fusions of selected candidate proteins were constructed as described elsewhere (Hruby et al, 2011). The constructs were integrated into the BY-SILAC strain expressing Shs1-mCherry under its native promoter.

For SPLIFF experiments, *SHS1* was cloned in frame with the CCG reporter (mCherry-C_{Ub}-GFP) in pRS306 (Sikorski & Hieter, 1989). The construct was integrated into the haploid JD47 strain (Madura et al, 1993) using the *URA3* gene as described (Moreno et al, 2013). Yeast strains for SPLIFF measurements were generated by mating of JD47 Shs1-CCG with JD53 (Dohmen et al, 1995) expressing a N_{ub}-fusion of interest under control of the copper-inducible promoter P_{CUP1}. The N_{ub}-fusion strains were constructed as described elsewhere (Hruby et al, 2011) .

For pulldown experiments, the indicated ORFs were cloned in frame with a TAP tag sequence into pRS306. The constructs were integrated into the haploid BY-SILAC strain using the *URA3* gene.

Settings for the MS searches

MS/MS data were searched against the *Saccharomyces* Genome Database (version of June 2012) using Andromeda (Cox & Mann, 2008). Searches were performed with tryptic specificity and a maximum of two missed cleavages. Oxidation of methionine and acetylation of protein N-termini were set as variable modifications; carbamidomethylation of cysteine residues was set as fixed modification. Mass spectra were searched using the default settings of Andromeda with mass tolerances for precursor and fragment ions of 6 ppm and 0.5 Da, respectively. Peptides and proteins were identified with a false discovery rate of < 1 %. At least one unique peptide with a minimum length of 6 (alpha-factor and hydroxyurea data) or 7 amino acids (*cdc15-1* data) was required. $^{13}C_6{}^{15}N_2$-lysine (Lys8) and $^{13}C_6{}^{15}N_4$-arginine (Arg10) were set as heavy labeled amino acids. Relative peptide and protein quantification was based on unique peptides and at least one ratio count (i.e. SILAC peptide pair). The options "requantify", "filter labeled amino acids", and "match between runs" were enabled. Low-scoring versions of identified peptides were not considered for quantification.

Compilation of excluded candidates

Hydroxyurea is known to influence the abundance and localization of 83 and 173 proteins, respectively (Tkach et al, 2012). All specific interaction partners of the septins in S-phase were manually screened for described changes in protein localization after HU-treatment. Hgh1 is known to change its localization after HU-treatment and becomes enriched in the nucleus. Further 27 proteins are described in literature to relocalize after HU-treatment from the bud neck to other compartments, especially the cytosol. These proteins, including some known physical septin interactors like Boi1 (Tonikian et al, 2009) and Gic1 (Iwase et al, 2006), are potential false negative interactors.

A shift of temperature to 37°C induces heat shock effects inside the yeast cell. The primary effect of a heat shock is protein unfolding, leading to an upregulation of chaperone expression. Known side effects are overexpression of proteins involved in respiration, transport, oxidation defense and metabolism (Gasch et al, 2000; Morano et al, 2012). We identified in the *cdc15-1* arrested cultures two heat shock proteins, Hsp12 and Hsp42, three proteins involved in metabolism (Gpd1, Lat1 and Pdx1) and Dcs2, a stress-induced regulatory protein, as specific interaction partners of the septins. The expression of Dcs2, Gpd1, Hsp12 and Hsp42 is known to be upregulated after heat shock (Gasch et al, 2000). These proteins were thus considered as experimental artifacts and were not further validated.

Steady state SPLIFF and quantitative analysis of SPLIFF data

For steady state SPLIFF, the microscopy settings were as following: The camera EM-gain was set to 200 and the laser settings were 35 % power, 100 ms exposure for the 488 nm laser and 50 % power, 250 ms exposure for the 561 nm laser, respectively. Images were acquired in a series of nine z-slices with an interval of 0.26 µm.

The quantitative analysis of the steady state SPLIFF data was performed as described before (Moreno et al, 2013) with some modifications

Acquired images were imported to ImageJ, all z-slices were projected to one layer and image channels were split into separate files. Regions of interest (ROIs) spanning the complete bud neck of cells with stable septin collar were selected. The mean intensity of the GFP- and mCherry-channel for each ROI was measured ($I_{bud\ neck}$) and the corresponding channel background ($I_{channel\ background}$) was subtracted (I).

$$\text{(I)} \qquad I_{bud\ neck} - I_{channel\ background} = rIb$$

The relative intensities at the bud neck (rIb) of both channels were used to determine the extent of the interaction (F_D) by calculating the conversion (i.e. cleaving and degradation of the GFP) of the CCG- to the CC-fusion (II).

$$\text{(II)} \qquad F_D = \frac{rIb_{RED} - rIb_{GREEN}}{rIb_{RED}}$$

The resulting F_Ds were normalized by subtraction of the F_D of a negative control ($F_{D\ Nub\text{-}empty}$) (III).

$$\text{(III)} \qquad F_D - F_{D\ Nub\text{-}empty} = nF_D$$

The complete conversion of the CCG- to the CC-fusion was set to 100% (IV).

$$\text{(IV)} \qquad 1 + \left|F_{D\ Nub\text{-}empty}\right| = 100\ \%$$

The calculated normalized F_Ds were plotted as vertical Scatter Plot. Single points represent independent nF_D-measurements of bud necks in different cells. Mean values and SEMs were calculated.

The quantitative analysis of the time-resolved SPLIFF data was performed essentially as described (Moreno et al. 2013) with one modification: The relative intensities were obtained by normalizing to the point of the lowest conversion of the respective curve (instead of normalizing to a point before mating).

Supplementary References

Cox J, Neuhauser N, Michalski A, Scheltema RA, Olsen JV, Mann M (2011) Andromeda: a peptide search engine integrated into the MaxQuant environment. J Proteome Res 10: 1794-1805

Dohmen RJ, Stappen R, McGrath JP, Forrova H, Kolarov J, Goffeau A, Varshavsky A (1995) An essential yeast gene encoding a homolog of ubiquitin-activating enzyme. J Biol Chem 270: 18099-109

Gasch AP, Spellman PT, Kao CM, Carmel-Harel O, Eisen MB, Storz G, Botstein D, Brown PO (2000) Genomic expression programs in the response of yeast cells to environmental changes. *Mol Biol Cell* 11:4241-4257

Hruby A, Zapatka M, Heucke S, Rieger L, Wu Y, Nussbaumer U, Timmermann S, Dunkler A, Johnsson N (2011) A constraint network of interactions: protein-protein interaction analysis of the yeast type II phosphatase Ptc1p and its adaptor protein Nbp2p. J Cell Sci 124: 35-46

Iwase M, Luo J, Nagaraj S, Longtine M, Kim HB, Haarer BK, Caruso C, Tong Z, Pringle JR, Bi E (2006) Role of a Cdc42p effector pathway in recruitment of the yeast septins to the presumptive bud site. *Mol Biol Cell* 17:1110-1125.

Madura K, Dohmen RJ, Varshavsky A (1993) N-recognin/Ubc2 interactions in the N-end rule pathway. J Biol Chem 268: 12046-54

Morano KA, Grant CM, Moye-Rowley WS (2012) The response to heat shock and oxidative stress in Saccharomyces cerevisiae. *Genetics* 190:1157-1195.

Moreno D, Neller J, Kestler HA, Kraus J, Dunkler A, Johnsson N (2013) A fluorescent reporter for mapping cellular protein-protein interactions in time and space. Mol Syst Biol 9: 647

Pereira G, Manson C, Grindlay J, Schiebel E (2002) Regulation of the Bfa1p-Bub2p complex at spindle pole bodies by the cell cycle phosphatase Cdc14p. J Cell Biol 157: 367-379

Rigaut G, Shevchenko A, Rutz B, Wilm M, Mann M, Seraphin B (1999) A generic protein purification method for protein complex characterization and proteome exploration. Nat Biotechnol 17: 1030-2

Sikorski RS, Hieter P (1989) A system of shuttle vectors and yeast host strains designed for efficient manipulation of DNA in Saccharomyces cerevisiae. Genetics 122: 19-27

Tkach JM, Yimit A, Lee AY, Riffle M, Costanzo M, Jaschob D, Hendry JA, Ou J, Moffat J, Boone C, Davis TN, Nislow C, Brown GW (2012) Dissecting DNA damage response pathways by analysing protein localization and abundance changes during DNA replication stress. *Nat Cell Biol* 14:966-976.

Tonikian R, Xin X, Toret CP, Gfeller D, Landgraf C, Panni S, Paoluzi S, Castagnoli L, Currell B, Seshagiri S, Yu H, Winsor B, Vidal M, Gerstein MB, Bader GD, Volkmer R, Cesareni G, Drubin DG, Kim PM, Sidhu SS, Boone C (2009) Bayesian modeling of the yeast SH3 domain interactome predicts spatiotemporal dynamics of endocytosis proteins. *PLoS Biol* 7:e1000218.
