# Supplementary material for: Identification of Cell Cycle Dependent Interaction Partners of the Septins by Quantitative Mass Spectrometry
Source: PLoS One. 2016 Feb 12;11(2):e0148340. doi: 10.1371/journal.pone.0148340 (PMC4752459; doi:10.1371/journal.pone.0148340)
Supplement: S2 Table — (PDF) [file pone.0148340.s010.pdf]

Table S 2 Steady-state SPLIFF analysis data

nF_D normalized extent of interaction; **SEM** standard error of the mean

N_{ub}-Fusion	0 Cu^{2+}		100 µM Cu^{2+}	
	Measured cells	nF_D ± SEM [%]	Measured cells	nF_D ± SEM [%]
Bni4	47	-3.39 ± 2.37	46	7.31 ± 2.00
Bni5	28	87.77 ± 0.77	20	93.52 ± 0.67
Bud3				
collar	30	59.20 ± 2.27	40	75.77 ± 1.28
split rings	30	68.38 ± 2.01	40	84.78 ± 0.77
Bud4				
collar	39	61.54 ± 1.95	40	90.63 ± 0.98
split rings	25	69.08 ± 2.60	31	93.11 ± 0.44
Cdc11	40	95.18 ± 0.61	40	93.99 ± 0.29
Coy1	18	8.32 ± 3.09	20	11.56 ± 4.10
Cse1	21	25.35 ± 4.07	25	83.20 ± 0.80
Gin4	41	86.76 ± 0.87	44	96.18 ± 0.15
Glc7	27	-10.41 ± 7.99	27	6.33 ± 8.44
Hsl1	42	55.21 ± 1.70	46	87.65 ± 0.84
Mrc1	28	33.94 ± 2.89	24	49.74 ±2.82
Nap1	30	2.47 ± 3.47	22	-40.11 ± 5.15
New1	18	46.59 ± 4.72	30	89.39 ± 0.54
Nop4	21	8.29 ± 3.36	23	61.84 ± 1.90
Nui-empty(P)	43	0.00 ± 1.92	43	0.00 ± 3.13
Ppz2	35	-15.86 ± 2.44	36	7.69 ± 3.37
Prk1	25	-18.73 ± 4.16	14	10.70 ± 4.88
Rlp7	25	25.41 ± 3.14	22	45.82 ± 3.12
Sfb3	30	20.63 ± 2.88	20	66.59 ± 3.36
Sic1	30	2.80 ± 3.28	27	10.43 ± 4.98
Sla2	28	17.88 ± 3.57	19	93.49 ± 0.63
Ste20	26	31.58 ± 1.91	20	62.53 ± 3.12
Syp1	26	34.48 ± 2.34	19	53.13 ± 3.54
Vps1	44	6.48 ± 2.65	21	50.72 ± 2.55
