# Supplementary material for: Identification of Cell Cycle Dependent Interaction Partners of the Septins by Quantitative Mass Spectrometry
Source: PLoS One. 2016 Feb 12;11(2):e0148340. doi: 10.1371/journal.pone.0148340 (PMC4752459; doi:10.1371/journal.pone.0148340)
Supplement: S3 Table — (PDF) [file pone.0148340.s011.pdf]

Table S3: Yeast strains used in this work.

Unlisted N_{ub}-fusion strains were available in the Johnsson Lab (Hruby et al, 2011).

Name	Relevant genotype	Source	Origin
BY Ammerer	*MAT*a *arg4::KanMX4 his3Δ1 leu2Δ0 lys1::KanMX4 lys2Δ0 ura3Δ0*		Ammerer Lab, MFPL Vienna University
BY SILAC	*TRP1::HIS3*	BY Ammerer	This work
Bud3-TAP	*BUD3::BUD3-TAP URA3*	BY SILAC	This work
Bud4-TAP	*BUD4::BUD4-TAP URA3*	BY SILAC	This work
Cdc11-GFP	*CDC11::CDC11-GFP TRP1*	BY SILAC	This work
Cdc11-TAP	*CDC11:CDC11-TAP K. lactis-TRP1*	BY SILAC	This work
BY SILAC *cdc15*ts	*CDC15::cdc15*-1 *C. maltosis-LEU2*	BY SILAC	This work
Cdc11-GFP	*CDC11::CDC11-GFP TRP1*	BY SILAC *cdc15ts*	This work
Cdc11-TAP	*CDC11::CDC11-TAP K. lactis-TRP1*	BY SILAC *cdc15ts*	This work
Shs1-mCherry Tub1-GFP	*SHS1::SHS1-mCherry URA3 TUB1::TUB1-GFP TRP1*	BY SILAC *cdc15*ts	This work
New1-TAP	*NEW1::NEW1-TAP URA3*	BY SILAC	This work
Pno1-TAP	*PNO1::PNO1-TAP URA3*	BY SILAC	This work
Rlp7-TAP	*RLP7::RLP7-TAP URA3*	BY SILAC	This work
Sfb3-TAP	*SFB3::SFB3-TAP URA3*	BY SILAC	This work
Shs1-mCherry Bni4-GFP	*SHS1::SHS1-mCherry URA3 BNI4::BNI4-GFP TRP1*	BY SILAC	This work
Shs1-mCherry Nip1-GFP	*SHS1::SHS1-mCherry URA3 NIP1::NIP1-GFP TRP1*	BY SILAC	This work
Shs1-mCherry Pno1-GFP	*SHS1::SHS1-mCherry URA3 PNO1::PNO1-GFP TRP1*	BY SILAC	This work
Sla2-TAP	*SLA2::SLA2-TAP URA3*	BY SILAC	This work
Ste20-TAP	*STE20::STE20-TAP URA3*	BY SILAC	This work
Syp1-TAP	*SYP1::SYP1-TAP URA3*	BY SILAC	This work
Vps1-GFP	*VPS1::VPS1-GFP TRP1*	BY SILAC	This work
ESM1278	*MAT*a *ade2-101 leu2Δ1 lys2-801 trp1-Δ63 his3-Δ200 ura3-52 cdc15-1*		(Perreira *et al.*, 2002)
ESM1278 *cdc15-1 LEU2*	*cdc15-1 C. maltosis-LEU2*	ESM1278	This work
JD47	*MAT*a *his3*-Δ200 *leu2-3,112 lys2-801 trp1*Δ63 *ura3-52*		(Madura *et al.*, 1993)
Cdc3-CCG	*CDC3::CDC3-CCG URA3*	JD47	This work

Cdc10-CCG	*CDC10::CDC10-CCG URA3*	JD47	This work
Cdc11-CCG	*CDC11::CDC11-CCG URA3*	JD47	This work
Cdc12-CCG	*CDC12::CDC12-CCG URA3*	JD47	This work
Shs1-CCG	*SHS1::SHS1-CCG URA3*	JD47	This work
Shs1-mCherry Bud3-GFP	*SHS1::SHS1-mCherry URA3 BUD3::BUD3-GFP TRP1*	JD47	This work
Shs1-mCherry Bud4-GFP	*SHS1::SHS1-mCherry URA3 BUD4::BUD4-GFP TRP1*	JD47	This work
JD53	*MATα his3-Δ200 leu2-3,112 lys2-801 trp1Δ63 ura3-52*		(Dohmen *et al.*, 1995)
N_{ub}-Coy1	P_{COY1}::kanMX6 $P_{CUP1}N_{ub}$-HA	JD53	This work
N_{ub}-Cse1	P_{CSE1}::kanMX6 $P_{CUP1}N_{ub}$-HA	JD53	This work
N_{ub}-Glc7	P_{GLC7}::kanMX6 $P_{CUP1}N_{ub}$-HA	JD53	This work
N_{ub}-Mrc1	P_{MRC1}::kanMX6 $P_{CUP1}N_{ub}$-HA	JD53	This work
N_{ub}-New1	P_{NEW1}::kanMX6 $P_{CUP1}N_{ub}$-HA	JD53	This work
N_{ub}-Nop4	P_{NOP4}::kanMX6 $P_{CUP1}N_{ub}$-HA	JD53	This work
N_{ub}-Rlp7	P_{RLP7}::kanMX6 $P_{CUP1}N_{ub}$-HA	JD53	This work
N_{ub}-Sfb3	P_{SFB3}::kanMX6 $P_{CUP1}N_{ub}$-HA	JD53	This work
N_{ub}-Sic1	P_{SIC1}::kanMX6 $P_{CUP1}N_{ub}$-HA	JD53	This work
N_{ub}-Vps1	P_{VPS1}::kanMX6 $P_{CUP1}N_{ub}$-HA	JD53	This work
